# Manipulating the sleep-wake cycle and circadian rhythms to improve clinical management of major depression

**DOI:** 10.1186/1741-7015-11-79

**Published:** 2013-03-22

**Authors:** Ian B Hickie, Sharon L Naismith, Rébecca Robillard, Elizabeth M Scott, Daniel F Hermens

**Affiliations:** 1 Clinical Research Unit, Brain & Mind Research Institute, University of Sydney, 100 Mallett St, Camperdown, NSW, 2050, Australia; 2 School of Medicine, The University of Notre Dame, 160 Oxford St, Darlinghurst, Sydney, NSW, 2010, Australia

**Keywords:** Major depression, Sleep-wake cycle, Circadian rhythms

## Abstract

**Background:**

Clinical psychiatry has always been limited by the lack of objective tests to substantiate diagnoses and a lack of specific treatments that target underlying pathophysiology. One area in which these twin failures has been most frustrating is major depression. Due to very considerable progress in the basic and clinical neurosciences of sleep-wake cycles and underlying circadian systems this situation is now rapidly changing.

**Discussion:**

The development of specific behavioral or pharmacological strategies that target these basic regulatory systems is driving renewed clinical interest. Here, we explore the extent to which objective tests of sleep-wake cycles and circadian function - namely, those that measure timing or synchrony of circadian-dependent physiology as well as daytime activity and nighttime sleep patterns - can be used to identify a sub-class of patients with major depression who have disturbed circadian profiles.

**Summary:**

Once this unique pathophysiology is characterized, a highly personalized treatment plan can be proposed and monitored. New treatments will now be designed and old treatments re-evaluated on the basis of their effects on objective measures of sleep-wake cycles, circadian rhythms and related metabolic systems.

## Background

Nowhere is the loss of confidence in the diagnostic processes in psychiatry more acute than it is in relation to the major depressive disorders [1,2]. This is reflected not only in the acrimonious debate about proposed changes to the Diagnostic and Statistical Manual of Mental Disorders (DSM)-5 [3], but also in the degree of professional discord [4] and the sustained social critique of the current concepts [5,6]. More profoundly from a therapeutic perspective, it has contributed to the withdrawal of major pharmaceutical industry support for new drug development [7].

This all occurs at a time when internationally there is widespread recognition of the premature death and disability attributable to mood disorders, reflecting their early age-of-onset, high population prevalence, chronicity, comorbidity with physical illness and the degree of resultant impairment [8-10]. To reduce that burden, earlier identification and enhanced long-term care of those who are at risk or are in the early phases of life threatening or chronic disorders has been prioritized [8,11-15].

However, this key ‘pre-emptive’ approach is compromised by poorly-validated and entirely descriptive diagnostic systems [11-15]. Further, these systems were based on the experiences of middle or older age cohorts with recurrent or persistent disorders. By contrast, one of the few concepts that can be supported neurobiologically is that early-onset major depression (that is, develops before age 25 years) is pathophysiologically distinct from late-onset major depression (that is, develops after the age of 50 years, and typically in association with other genetic or vascular risk factors [16-19]).

When the current criteria are used as the basis for identifying biomarkers of a risk or illness course in very mixed clinical populations of those experiencing major depression, they result in very poor specificity. Previous attempts to link major depression to dysregulation of the hypothalamic-pituitary (HPA) axis were abandoned for this reason [20,21]. In parallel, there has been a failure to link major depression to any clear set of genetic risk factors [22]. Most importantly clinically, the outcome of current treatment trials is highly compromised. The examination of very heterogeneous groups of subjects, and particularly the inclusion of those with lower levels of severity of illness, appears to contribute substantially to the general failure to identify specific biomarkers and the large differences between active and placebo therapies [23-25]. Depression treatment is also sub-optimal due to the general lack of rapid onset of action. The risk of suicide or other self-harm remains high during the period of acute depression and only decreases substantially in parallel with the provision of effective treatments [26].

While various phenotypically-defined subgroups (for example, severe, melancholia, anxious or psychotic depression) have been proposed historically, each subtype has achieved only limited success against the key validating principles of specific genetic or environmental risk factors, discrete pathophysiological pathways or unique patterns of response to treatment. Instead, much clinical and epidemiological innovation has switched to identifying clearer points of illness onset and subsequent developmental paths, particularly those that occur from early adolescence through to early adulthood. While this approach was first utilized for psychotic disorders [27-29], current efforts now also focus on applying these techniques to the major mood disorders [30-33].

Marked interdisciplinary progress in the clinical and basic neurosciences of sleep-wake cycles and underpinning circadian systems has opened the door to a new way of conceptualizing at least a significant subpopulation of those who present with major mood disorders. The role that disruption of sleep-wake and circadian systems play in the risk of onset, early course, comorbidity, recurrence, persistence and the physical health complications of major mood disorders is being rapidly elaborated [34-38]. For example, our group has reported that short or excessively long sleep duration is associated with the onset and persistence of psychological distress in a sample of 20,822 people aged 17 to 24 years [39]. Key findings also come from experimental studies in healthy adults showing that delaying sleep onset or disturbing circadian synchrony causes profound changes in mood, cognitive function, motor activity and subjective symptoms of energy and well-being [40,41]. A growing body of studies conducted in subjects with other psychiatric illnesses (for example, psychotic disorders) also highlights associations between sleep-wake and circadian disruptions, concurrent mood disturbances and metabolic and other physical health complications [42-45].

This paper provides an overview of the extent to which current self-report and objective measures of sleep-wake cycles and circadian systems can be used to characterize individuals presenting with major mood disorders. It also considers how such clinical information can be used to inform choice of specific behavioral or pharmacological regimes and to monitor short and longer-term responses to treatment.

## Discussion

### Basic science developments in relation to the circadian clock

**Figure 1:**
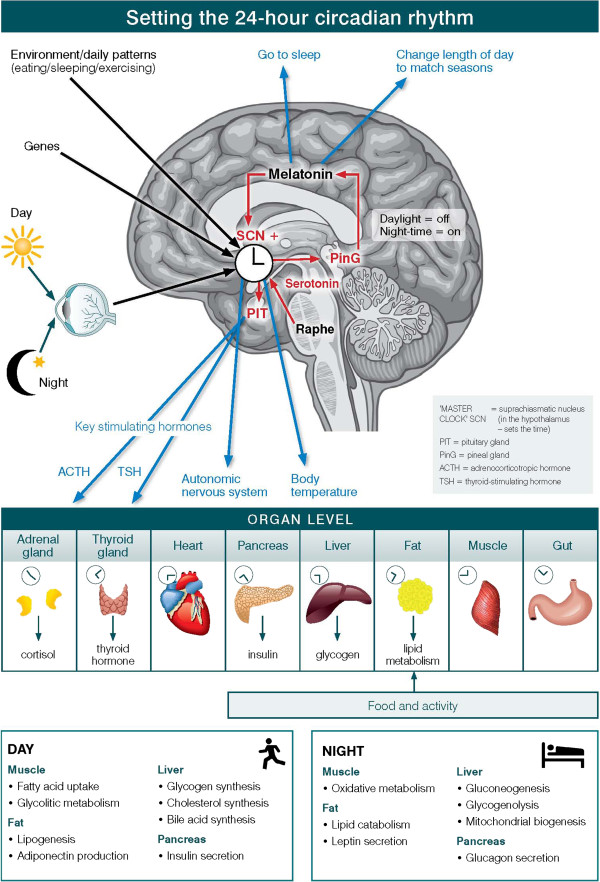
The master circadian clock in the human brain.

Circadian systems need to be considered in relation to three differing levels of organization of information and operation (see Figure 1). First is the way in which the physical environment communicates (or ‘Inputs’) key information, particularly related to differentiation of night from day, to the internal ‘master’ clock (located in the brain’s suprachiasmatic nucleus (SCN)). Second are the ‘Intrinsic’ brain factors, consisting of the master clock and its linked regulatory systems (notably secretion of melatonin from the pineal gland). These contribute to sleep onset, sleep architecture, sleep-wake cycles and other central nervous system (CNS)-dependent behavioral changes. Third is the way in which the circadian system coordinates all other hormonal, metabolic, immune, thermoregulatory, autonomic nervous and other physiological processes to optimize the relationships between behavior and body functions (that is, the ‘Outputs’).

At the cellular level, almost all individual cells and, hence, organ systems have their own intrinsic clocks. As these cellular (for example, fibroblasts, fat cells, muscles) and organ-based (for example, liver, pancreas, gut) clocks run to intrinsically different period lengths, the differing physiological systems need to be aligned in coherent patterns. Fundamentally, the master circadian clock permits the organism to align key behavioral and intrinsic physiological rhythms optimally to the external 24-hour light–dark cycle [46].

#### Molecular regulators of the brain clock

The brain’s master clock (in the SCN) is a small cluster of pacemaker neurons in the anterior hypothalamus. It operates a complex auto-regulatory network, utilizing a set of transcriptional activators and repressors [47-49]. These processes are under the control of a core set of regulatory genes including: *Period* – *Per1*, *Per2* and *Per3*; *Cryptochrome* – *Cry1* and *Cry2*; *Nuclear receptor subfamily 1* – *Nr1d1* (*REV-ERB-α*) and *Nr1f2* (*ROR-β*); *Circadian Locomotor Output Cycles Kaput* – *CLOCK*; and *Aryl hydrocarbon receptor nuclear translocator-like* – *Bmal1* and *Bmal2*. The transcription of *PER* and *CRY* genes is activated by the binding of *BMAL1-CLOCK* or *BMAL1-NPAS2*. The expression of *PER* and *CRY* genes is rhythmic and is highest early in the day. Light exposure also drives the *PER1* and *PER2* expression genes. The progressive accumulation of the proteins that result from this activated transcription, over the daily period, eventually feeds back (via the formation of *Per*-*Cry* complexes) to reduce their own transcription (the so-called ‘hour-glass’ effect). The strong correlation between gene expression and circadian transcription factor binding leads to genome-wide circadian rhythms [50]. Across the past decades, it has been well established that the disruption of various components of the molecular clock leads to significant changes in circadian phenotype [51,52].

This 24-hour self-regulatory process is also influenced by a much wider set of small molecule modifiers, other genetic factors and transcriptional repressors (for example, deleted in esophageal cancer 1 - *Dec1* and *Dec2*) [53]. There are key proteins that impact on timing of the clock (through phosphorylation) including *casein kinase* (*CK*) 1 delta and epsilon (*Csnk1d* and *Csnk1e*). Selective inhibition studies indicate that *Csnk1d* appears to be a predominant mediator of circadian timing [54-56]. A variety of other protein kinases and phosphatases can also impact on circadian timing. The expansion of animal models (particularly in mice and zebrafish [57,58]), utilizing genetic manipulation or new environmental or developmental models, has created important new tools for further unraveling the complexity of these processes.

#### Glycogen synthase kinase 3 beta (GSK-3β) as a therapeutic target

*Glycogen synthase kinase 3 beta* (*GSK-3*β) plays a key regulatory role in circadian rhythms as it phosphorylates the mammalian *Per2* protein, facilitating nuclear entry - a key step in the auto-feedback loop. It is now more than 60 years since the anti-manic effect of lithium was first reported [59]. A key clinical observation relates to its calming effect without causing overt sedation. Another key aspect is that its main therapeutic value is in the longer-term reduction in symptoms of mania and prevention of relapse. Recently, the inhibitory effects of lithium on *GSK-3*β have been described [60]. However, there is ongoing debate as to whether the characteristic period-lengthening effects of lithium may actually be mediated by mechanisms other than inhibition of *GSK-3*β [58]. Genetic variations in *GSK-3*β have also been linked with the risk to depressive disorders, and most notably to bipolar disorder [61]. Hence, *GSK-3*β is one of a number of key targets being explored with regards to possible new agents for circadian-related sleep-wake or mood disorders [62-64].

#### Circadian function and human health

The adverse health effects of a disturbed circadian system are now under very active investigation. The major driver behind this increased focus on the 24-hour sleep-wake cycle and related circadian rhythms in clinical medicine has been the rapid progress in our understanding of the downstream effects on neurohormonal, metabolic, autonomic nervous and immune functions (see Figure 1). In humans, the aggregate outputs of the circadian system depend principally on the ways in which the brain, operating under genetic, behavioral (for example, eating behavior, physical activity), as well as other external (for example, light exposure, seasonality) and internal (for example, other sleep onset vs. awakening systems) cues, controls the central timing of circadian rhythms [47,65-67].

The rhythmic expression of the core clock genes operates on a period slightly longer than 24-hours, unless it is modified or ‘entrained’ by key environmental inputs (in humans it is most notably the daily light–dark cycle). Importantly, the rhythmic and circadian output of the whole process has been remarkably preserved across species, indicating its fundamental importance to survival. As animals age it would appear that the combination of strong circadian rhythms and caloric restriction imparts additional advantages.

#### Inputs to the brain clock

The input of light signals from the eye to the SCN is critical to setting the circadian period to a 24-hour cycle. Changing light signals indicate not only the transitions from day to night to day but also the change in day length that occurs across seasons. Light signals are conveyed by a monosynaptic pathway (that is, the retino-hypothalamic tract) from the retinal ganglion cells (which contain the photopigment melanopsin) to the cells of the SCN (via a glutamatergic signal). Additionally, the SCN receives an important serotoninergic input from the median raphe nucleus (that is, via non-photic stimuli). Hence, those behaviors or disorders that are associated with changes in serotoninergic mechanisms (as well as compounds that alter serotonin concentrations) may also have important impacts on circadian-dependent phenomena. The serotoninergic, non-photic inputs and the glutamatergic, photic-dependent signals have opposite effects at the level of the SCN.

With regards to external cues, it is clear that in humans the daily timing of a range of behaviors have an important influence on circadian rhythms [67]. These include: (i) awakening and rising from bed; (ii) light exposure, particularly to light that is in the ‘blue’ spectra (which is most efficient in suppressing melatonin secretion) [68,69]); (iii) physical activity and exercise, with additional effects of duration and intensity; (iv) eating patterns; (v) social and occupational activities; and, (vi) nighttime activity and related time of sleep onset.

#### ‘Morningness’ vs. ‘eveningness’

In humans, there is considerable inter-individual variability in the circadian phase, as well as variation in the peak periods of key behavioral (for example, mood, concentration, energy), neurohormonal (for example, cortisol peak, regulation of thyroid hormone) and body temperature elements. This intrinsic variability, which appears to be largely under genetic control, can be measured by self-report along a dimension of ‘morningness’ vs. ‘eveningness’ in the preferred timing for regular sleep schedules and other daily activities. Importantly, the tendency towards ‘eveningness’ has been associated with higher depressive symptoms [70,71]. In twin studies of bipolar disorder, affected individuals (compared with their co-twin) show greater seasonal changes in sleep patterns and mood, consistent with the extent to which environmental influences on the circadian systems are, in part, mediated by differences in genetic sensitivity [72].

#### The outputs of the brain clock

The outputs of the SCN exert their influence on other key regulatory systems; first, in the brain (see the critical SCN-pineal/melatonin loop, Figure 1) and, second, on other major physiological systems (via their impacts on the pituitary and linked neurohormonal systems, thermoregulation, the autonomic nervous system and other signaling mechanisms). The key physiological result of these inter-linked processes is coordination of the internal environment so that the various hormonal, immune and metabolic factors are in synchrony with each other and, critically, with the behavior of the individual (for example, eating, sleeping, exercising).

Lesion-based experiments have indicated that what is lost through destruction of the SCN is not the circadian system itself but this central coordinating role. Genetic alterations of clock genes in mice indicate the extent to which the coordination of key behavioral factors is disturbed. For example, mice with a point mutation in the *CLOCK* gene display: (i) hyperactivity over the light/dark cycle;, (ii) reduced depression-like behavior in rodent testing paradigms;, and, (iii) an increase in reward value in response to self-stimulation tests [73-75]. Alterations of clock genes also increase dopamine release, enhance sensitivity to dopamine receptor agonists, and alter the relative distribution of D1 and D2 receptors [76]. Moreover, *Per1-2* homolog dysfunctions in mice cause an increase in anxiety-like behavior and, conversely, a mouse model of depression leads to a reduction in *Per1-2* expression [77]. Accordingly, in humans, a growing body of evidence is linking various components of the molecular circadian clock to various health conditions, including mental disorders (for reviews, see [78,79]).

#### Melatonin and communication via the pineal gland

From a circadian perspective, one of the principal outputs of the SCN is communication with the pineal gland, which controls the daily pattern of melatonin release [80] (Figure 1). Again, this is a 24-hour feedback system, whereby light exposure (through the eye and signaled via the SCN) strongly inhibits melatonin release during daylight hours. This is followed by a rapid rise in melatonin secretion about two to three hours before habitual sleep onset (typically mid-evening). Melatonin has a wide range of CNS (including the SCN) and peripheral targets. Specifically, melatonin induces sleep onset, promotes optimal sleep architecture and coordinates other physiological and behavioral aspects of the sleep period. In turn, melatonin feeds back directly onto the SCN to inhibit the circadian signal for increasing wakefulness. It also competes with the activating effects of serotoninergic inputs to the SCN. In this way, endogenous melatonin (or the appropriate use of exogenous melatonin at the right point in the daily cycle) plays a key role in stabilization of daily rhythms. Some of these chronobiotic effects seem to be modulated by the interactions between melatonin and clock genes [81].

The other key outputs of the SCN (acting initially via the paraventricular nucleus; PVN) include regulation of the pituitary release of key stimulating hormones and autonomic neurons (see Figure 1). These have their impacts on a wide range of peripheral organs. Most notably, from a circadian perspective, adrenocorticotropic hormone (ACTH) regulation of cortisol release from the adrenal glands is central to the coordination of brain-dependent mechanisms and peripheral physiology. In recent years, there has also been much greater focus on the extent to which other key metabolic functions in the liver and pancreas have their own intrinsic circadian patterns [82-86]. Importantly, food intake entrains the liver’s clock. Ideally, these metabolic patterns should be coordinated with signals from the SCN so that key aspects of feeding behavior, glucose control and fat regulation are optimally coordinated [87]. Recent animal studies indicate that synthetic *REV-ERB-a/b* agonists alter the circadian timing of clock genes’ expression in the hypothalamus and locomotor activity, while enhancing energy expenditure and reducing fat, triglycerides and cholesterol levels [88].

#### Homeostatic sleep processes

Importantly, circadian processes are one of two major regulatory systems influencing the daily sleep-wake cycle [89]. The other is a homeostatic process directly related to wake duration; the longer the period awake, the greater the accumulated ‘pressure’ to sleep again (that is, sleep debt). While accumulated sleep pressure, in combination with nighttime melatonin release drives sleep onset, the circadian system is seen to drive the strength and rhythmicity of daytime wakefulness. The homeostatic sleep pressure component drives slow wave (or deep) sleep (SWS) while rapid eye movement (REM) sleep is strongly influenced by the circadian component (interestingly, it was once assumed that all effective antidepressants shared a capacity to suppress REM sleep). However, the optimal coordination of both of these mechanisms results in the maximal consolidation of sleep at night and wakefulness during the day.

#### The hypocretin/orexin systems

Another important aspect is the potential role of hypocretin/orexin, a key neuropeptide that also plays a central role in coordination of sleep-wake activity [90]. The timing of normal hypocretin/orexin production is critical as it contributes to staying awake (in humans typically later in the day) as sleep debt increases. In both humans and animals, absolute orexin deficiency is linked to the narcolepsy-cataplexy syndrome [91,92], while in animals a lack of orexin signaling increases risk of obesity [93]. Novel orexin antagonists have been developed for the treatment of primary or chronic insomnia and are undergoing extensive clinical trialing [94-96]. Consequently, further manipulation of hypocretin/orexin signaling is now under consideration as another pathway by which sleep, circadian and disturbed metabolic functions may all be enhanced [87,97].

In summary, we now have much more detailed knowledge of the ways in which the brain responds to a range of key environmental cues as well as the processes by which it, in turn, regulates the many other aspects of internal physiology that demonstrate specific rhythmic patterns (for example, hormonal, immunological and metabolic systems) [88,98-105].

### Clinical science developments in relation to the circadian clock

In the past, the traditional organ or clinical specialist-based approaches to clinical medicine have failed to take into account the health effects of important daily and seasonal fluctuations in key regulatory systems, such as the 24-hour sleep and circadian cycles. However, alongside the detailing of the molecular mechanisms of the clock and its signaling systems, the clinical applications of this new knowledge are beginning to be recognized. Twenty-four-hour sleep-wake and circadian cycles are now of great interest not only in sleep medicine and clinical psychiatry but also in aging and dementia, developmental and neurological disorders, obesity, diabetes and related metabolic disorders, immunology and infectious diseases, and cancer care. While in psychiatry generally, there has not yet been an effective translation of knowledge into clinical practice, this process has begun recently in the field of depressive disorders.

### The epidemiology and sub-classification of major depression

Historically, various sub-classifications for major depression have been proposed. Typically, each has prioritized different phenotypic characteristics (for example, severity, psychotic features, psychomotor change, presence of manic or hypomanic episodes, somatic or ‘atypical’ features) [16]. Although the more severe forms (for example, depression, bipolar disorder, melancholia, atypical and psychotic depression) implied a preferential response to biomedical therapies, only the psychotic forms demonstrate robust differences between active physical and placebo treatments [16]. To date, none of the phenotypically-derived sub-types have been clearly linked to a discrete pathophysiology. Further, no clear pattern of specific illness markers has been identified to aid diagnostic specificity or act as a useful proxy for personalized treatment selection or responsiveness.

While the 1990s saw the start of two decades of intensive genomic and brain imaging studies, the 1980s had seen the groundwork laid for three decades of population-based epidemiology and the much broader recruitment of subjects with less severe, persistent or recurrent disorders to clinical trials. Using standardized criteria, these studies identified large numbers of people in the broader community who met criteria for major depressive episodes [106]. In these broader populations, the large differences between active and placebo treatments evaporated and the differences between various physical and psychological approaches were minimized (see [1,2]).

The unintended consequences of these movements have been profound. On the one hand, it has led to a sustained critique by mental health specialists who discount the application of the clinical diagnosis of major depression in the wider population and, hence, call for a return to the earlier diagnostic systems that were derived from more severely or persistently-ill populations. Conversely, those more accustomed to primary care or population-based health perspectives sought to work with the new epidemiology, and, particularly, those key longitudinal studies from childhood or adolescence through to adulthood. These have also been enhanced by genetically-informative family and twin studies [107-109] as well as relevant risk factor studies [39]. This developmental perspective has generated new hypotheses about lifetime pathways (and underlying pathophysiologies) to adult depressive disorders [110,111].

#### Early-onset depressive disorders

The one key differentiation supported by genetic and environmental risk factor research, longitudinal and treatment studies and extensive concurrent neuroimaging and neuropsychological studies is the demarcation between early (that is, less than 25 years of age) and late-onset (typically after 50 or 60 years of age) depressive disorders [18,111,112]. Late-onset depression has its own clear set of vascular and other neurodegenerative risk factors [18,113-116] and represents one of the few psychiatric disorders where there is now a clear pathophysiological model (that is now being used to underpin population-based prevention and early intervention as well as clinical practice) [19,117-120].

**Figure 2:**
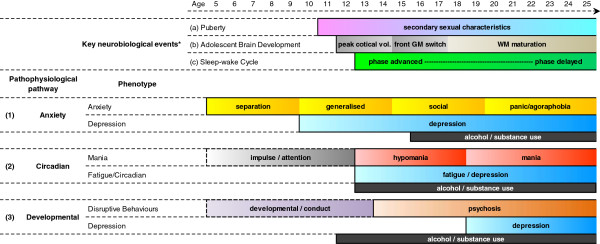
**Pathophysiological pathways to early-onset depressive disorders.** There are at least three common trajectories that lead to depression in the teenage and early-adult years. These are characterized by (**1**) ‘anxiety-central nervous system reactivity’, (**2**) ‘circadian and 24-hour sleep-wake cycle dysfunction’, and (**3**) ‘developmental brain abnormalities’. The six corresponding phenotypic patterns have distinct ages of onset and characteristics. From age 8 to 10 years onwards these processes are transformed by key neurobiological phenomena: (**a**) puberty, (**b**) adolescent brain development, and (**c**) sleep-wake cycle [see [122].

It is for those with early-onset depression, however, that new insights are desperately required. A new wave of clinical studies is focused on younger people and within those cohorts at least three clear trajectories of illness are increasingly apparent (see Figure 2). The most common would appear to be that dominated by child and adolescent anxiety, with the onset of more discrete depressive symptoms in early and mid-adolescence. It is this common form which offers perhaps the greatest hope for broadly-applied prevention and early interventions utilizing well-founded cognitive and behavioral strategies [121]. The second, and largely ignored by the traditional mood disorders literature, is that related to other childhood onset and adolescent-related conduct, behavioral and attention disorders. The third appears to be a pattern of mood disorders which may well be characterized better as disorders of energy or activity due to underlying perturbations of sleep-wake cycles and linked circadian rhythms. This last type is the subject of the remainder of this paper.

### Mood disorders and sleep-wake and circadian cycles

As the debate about the pathophysiology of mood disorders has focused primarily on the assumption that the core pathology(ies) must relate to disordered emotion, other features such as sleep-wake cycle and circadian changes have been downplayed. Most typically, major depressive disorders (see Table 1) have been associated with a wide range of perturbations of sleep and circadian physiology. Up to 60% of young persons with depressive disorders run an illness course characterized by marked delays in the sleep-wake schedule [123], and these fluctuations are often accompanied by changes in subjective energy, cognitive functions and mood. Very recent epidemiological evidence emphasizes that this pattern is clear from the onset in early to mid-adolescence and, contrary to previous dogma, does not develop solely in the early or mid-adult period [124]. Other evidence suggests the extent to which this pattern of low energy, as distinct from depressed mood, has its own set of genetic and environmental determinants in adolescence [109], as well as in mid- or later-life [125-130].

**Table 1:** Characteristic sleep-wake and circadian features of selected mood disorders

**Mood syndromes**	**Sleep-wake and circadian features**
Major depression	● Subjective sleep-wake complaints (often preceding the onset or recurrence of depressive episodes)
	Difficulty falling asleep, staying asleep or early morning awakening [131,132]
	Disturbing dreams [133]
	Unrefreshing shallow sleep [132,134]
	Daytime fatigue and sleepiness [132,134,135]
	● Macro and microarchitecture of sleep
	Abnormal sleep duration [136]
	Prolonged sleep onset latency [136]
	Shortened REM latency and increased rapid eye movements [136-140]
	Increased sleep fragmentation [136,138]
	Decreased SWS and increased REM sleep and (especially in the first sleep cycle) [136,140]
	Reduced slow wave activity and number of slow waves [136]
	High comorbidity with sleep-related breathing disorders [141,142]
	● Biological rhythms
	Abnormal sleep phase [71, 143 ]
	Reduced melatonin secretion [144-149]
	Increased 24-hour levels and variability of cortisol secretion [145,150,151]
	Reduced circadian amplitude and increased nighttime body temperature [147,151,152]
	Reduced heart rate circadian amplitude [153-155]
	Depressive symptoms associated with increased nocturnal blood pressure in males
	Abnormal cytokines, neurotransmitters, endocrine (for example, melatonin, cortisol, thyrotropin) and neuroimmune circadian rhythms [97,144-148,151,156-168]
	Abnormal circadian mood variations [157,159,169-171]
	Possible seasonal variations (not exclusive to seasonal affective disorder) [172]
	● Increased depressive symptoms are associated with more pronounced misalignment between melatonin, temperature and sleep-wake rhythms [173]
Depression in youth	● Subjective sleep-wake complaints
	Difficulty falling asleep and staying asleep [174]
	Difficulty waking up in the morning [175]
	● Macro and microarchitecture of sleep
	Lower intra- and inter-hemispheric coherence in delta and beta activity, especially in girls [176,177]
	Otherwise similar features to those seen in adult depression, but expressed to a lesser degree [177-184]
	● Biological rhythms
	Higher levels of ‘eveningness’ preference [70]
	Lower circadian amplitude [185]
	Delayed sleep phase and melatonin onset, especially in those with bipolar disorder [123,212]
	Elevated evening/nighttime cortisol levels [186,187]
Late-life depression	● Macro and microarchitecture of sleep
	Lower increase in REM sleep duration [136]
	Otherwise similar features to that seen in adult depression, but more pronounced [136]
	● Biological rhythms
	Increased early morning awakenings [188]
	Abnormal melatonin levels [189]
	High prevalence of abnormal blood pressure circadian rhythms [190]
	● Sleep and circadian disturbances have been associated with cognitive decline, relapses and mood deterioration [43,116]
Bipolar disorders	● Characterized by episodic periods of sleep loss (that is, switching from wake to normal duration sleep state and back again over a 24-hour period) [191,192]
	● High prevalence of hypersomnia, especially ‘morning hypersomnia’ [193-195]
	● Insomnia often occurring before and during manic episodes [196]
	● Insomnia or hypersomnia often occurring before and during depressive episodes [196]
	● Macro and microarchitecture of sleep
	Depressive phase: longer sleep onset latency and greater REM fragmentation, but otherwise similar to people with unipolar depression [197-200]
	Manic phase: Prolonged sleep onset latency, decreased sleep duration, lower sleep efficiency, shortened REM sleep latency and increased REM activity and density [201,202]
	● Disturbed biological rhythms
	Short circadian period [203]
	Possible hypersensitivity to light suppression of melatonin [204,205]
	Diurnal variations in the direction of mood cycle switch [206]
	Possibly enhanced disturbances in thyrotropin rhythms [207]
	Onset of mania episodes at key points in seasonal transitions [72,208]
	High prevalence of evening chronotypes (that is, preference for late bed and wake times) and late sleep onset, especially in younger individuals [209,210]
	In youth, the delay in sleep-wake cycles and dim light melatonin onset (DLMO) is more pronounced than what is seen in unipolar depression [123,211]
Seasonal affective disorder	● Disrupted sleep
	Hypersomnia (typically in winter-onset) [212-214]
	Insomnia (typically in summer-onset) [212,214]
	● Disrupted biological rhythms
	Delayed melatonin, cortisol and temperature rhythms [215-217]
	Seasonal pattern of changes in symptoms [218]
	Increased sensitivity of melatonin to light in the winter and decreased sensitivity in the summer [219]
Dysthymia	● Similar features to that seen in major depression expressed to a lesser degree [220]

Key features, such as onset of illness episodes, in relation to changes in sleep patterns and/or season have not necessarily been emphasized. For some patient subgroups these involve characteristic shifts into or out of periods of prolonged depression, oversleeping, low energy and apathy. For others (notably those on the bipolar spectrum), this may also result in discrete periods of decreased sleep, high energy, increased activity and irritability or excitement. Other indicators of disturbed circadian rhythm are observable in patients with major depression including changes in the normal patterns of diurnal mood variation or loss of normal daily variations in body temperature (with elevated mean nocturnal temperatures resulting in the perception of nighttime ‘sweats’). The possible role of the circadian clock in the pathophysiology of depression is also supported by post-mortem histological anomalies in the SCN of depressed individuals [221].

#### Typical and atypical forms of depression

A large number of sleep or circadian abnormalities have been documented across the various forms of major mood disorders (see Table 1). In some forms of depression, notably those ‘typical’ or ‘melancholic’ forms associated with early morning wakening and marked diurnal mood variation (often occurring in older rather than younger subjects), there may also be a marked change in the normal circadian variation in motor activity, with a shift towards shorter and more disturbed sleep and a phase advanced pattern. That is, the end result is being awake and potentially more agitated in the early hours of the morning. Overall, however, such forms of depression are associated with psychomotor slowing and a reduction in total daytime activity [222-226]. These severe forms of depression are also typically associated with decreased appetite and weight loss.

By contrast, ‘atypical’ forms of depression, which are often seen in teenage and young adult patients, are associated with later sleep onset, later sleep offset, prolonged rather than shortened sleep, daytime tiredness and fatigue as well as loss of motor activity in the mornings. These disorders are often associated with overeating, weight gain and increased risk of metabolic dysfunction. These patterns suggest underlying phase delay in the circadian system.

#### Loss of circadian hormonal patterns in depressive disorders

The symptoms of circadian disturbance seen in subgroups of depressive disorders are also accompanied by observable changes in the normal circadian patterns of release of ACTH (regulating cortisol release) and thyroid-stimulating hormone (TSH; regulating thyroid hormone release). The typical morning peak of cortisol release is normally well matched to the associated feelings of energy and our capacity to cope with physical stressors. The resulting altered patterns of cortisol release are generally consistent with underlying phase advance or loss of synchrony with other elements of the daily cycle. Changes in such key patterns of cortisol release appear to correlate also with daytime feelings of lack of energy and fatigue. Similarly, while there is evidence of changes in the amplitude and timing of the TSH release pattern, it would also generally suggest a pattern of phase advance.

#### Common genetic factors in circadian systems and risk of mood disorders

Of additional importance to the increased interest in sleep-wake cycles and circadian rhythms and risk of mood disorders, has been the recognition of the commonality between genetic determinants of circadian functions and those genes which may increase risk of mood disorders, particularly bipolar disorder [227]. While further studies are required, so far, variations of *Per3* have been associated with age of onset of mood disorders, response to treatment and circadian mood variations [228] and *Per 3*, *Bmal1*, *CLOCK*, *Nr1d1* and *Nr1f2* have all been linked to bipolar disorder [229-234]. Another related set of studies has begun to investigate whether the same genetic factors that predict response to antidepressant medicines also predict response to specific circadian-based therapies, such as light therapy or sleep deprivation [235].

#### Disturbances in melatonin secretion in mood disorders

In those with major depressive disorders, disturbances in the amplitude and rhythm of melatonin secretion have been described [see Table 1]. These are similar to those described in a range of other sleep or neuropsychiatric disorders associated with disturbed circadian function. Although phase advance and phase delay in melatonin secretion have both been reported, phase advance (largely in middle age and older cohorts) may be more typical. Additionally, changes in the rate of rise of evening melatonin and the amplitude of the melatonin response have been associated with various mood disorders. The findings with regards to phase advance versus phase delay for melatonin, cortisol and other circadian parameters in patients with major depression may be strongly influenced by the age of the subjects. Various relationships have been reported between the severity of these changes in timing or amplitude and reported severity of depression or degree of sleep disturbance. For those with bipolar disorder, there have been distinctly different patterns of melatonin secretion reported during the manic (phase advanced) as compared with the depressive phase (phase delayed).

For those with major depressive disorders, the role of sleep-wake cycles or circadian perturbations may be quite different at various life stages. For the early-onset group, it is likely that sleep-wake and circadian disturbances represent one discrete pathway to disorder; while for those with the late-onset sub-type, sleep-wake disturbance may be linked with concomitant structural and functional brain changes due to underlying vascular or neurodegenerative processes (see review by [19,236-238]).

#### The challenge of bipolar depression

One of the areas of greatest therapeutic frustration has been the treatment of the depressive phase of bipolar disorder. Importantly, conventional monoamine-based medications are of least benefit in these patients. At this time a new range of second generation antipsychotic medications, anticonvulsants and other mood-stabilizing agents form the basis of care (often in combination with antidepressant agents). Conventional lithium therapy is not an effective monotherapy for bipolar depression. Given the very strong circadian features of bipolar disorder, and this failure to develop effective therapies for the depressive phase based on monoamine targets, it (along with the other circadian-like mood disorders such as seasonal affective disorder, winter depression, seasonally-sensitive unipolar depression, bipolar spectrum disorders, atypical depression) may benefit from a new circadian-based approach to treatment selection. A recent report suggests that adjunctive use of a novel melatonin agonist (ramelteon) may help to prevent relapse of bipolar disorder, and most notably relapse into the depressive phase [239].

#### The importance of internal desynchrony

**Figure 3:**
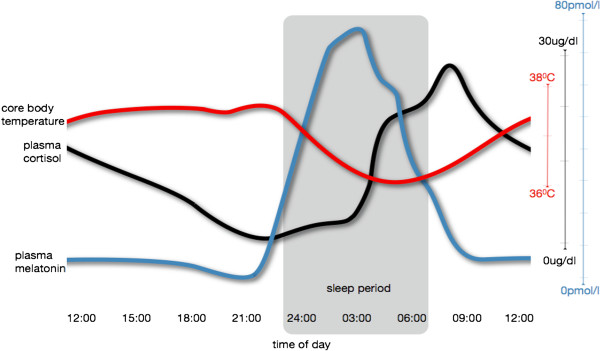
The normal synchronous relationships between sleep and daytime activity and cortisol, melatonin and body temperature.

Various authors (see [34,35] for review) have proposed that what may be most characteristic of the neuropsychiatric disorders is the extent to which they result in ‘desynchrony’, that is, both loss of synchrony between the key behavioral and physiological events as well (see Figure 3) as loss of synchrony between the various physiological events (that is, in the CNS itself and the key peripheral systems). Many human studies of dysregulation of central hormones (for example, HPA axis), or immune function, in those with major depression and a range of other neuropsychiatric disorders have moved their emphasis away from recording absolute changes in serum levels to evaluating putative changes in relation to deviations from normal circadian patterns [146,151,165,167,168,240].

Major depression appears to be characterized by loss of synchrony between the different sleep-wake, mood, cognitive, motor, hormonal, immune, thermoregulatory and/or metabolic aspects of the normal circadian rhythm [35]. In addition to its detrimental effects on the sleep-wake cycle, this internal desynchrony may well account for the range of somatic symptoms (fatigue, temperature dysregulation, muscle pain) and other physiological abnormalities that have been linked to various forms of major depression. While it has previously been assumed that circadian signaling mechanisms from the brain to the periphery (see Figure 1) are maintained largely by hormonal signals, recent evidence suggests a key role of daily variations in body temperature [241].

It is tempting to postulate that the blunted amplitude of the temperature rhythm commonly found in patients with major depression could be a major driver of internal desynchronization, and, hence, of many of the somatic symptoms of depression as well as some of its metabolic and immunological complications. If this were so, restoration of the normal variation in body temperature across the 24-hour cycle would be a key therapeutic target. This may be manipulated by strategies designed to increase body temperature in daylight hours (particularly early morning – for example, with vigorous exercise) or decrease body temperature in the evening (that is, through environmental or pharmacological manipulations).

### Clinical studies of young people with major depressive disorders

#### Monitoring the 24-hour sleep-wake and activity cycle

A key aspect of those studies that focus on disruptions of sleep-wake cycles and circadian rhythms is the capacity to integrate a number of new detailed objective measures (Table 2). These complement extended self-report measures of daily fluctuations in sleep, mood, energy or physical activity. They include extended periods of actigraphy (for example, typically at least two weeks), as well as 24- to 72-hour assays of body temperature and 24-hour fluctuations in cortisol or melatonin (including under controlled environmental parameters, such as light exposure). Even though actigraphy measures are indirect and somewhat more variable (at least in the short term) than other circadian markers, because of other behavioral and homeostatic influences, they have consistently been found to correlate with the circadian rhythm of endogenous melatonin and body temperature [242-249]. Other new technologies, such as smart phone applications, permit home monitoring of extended periods of sleep-wake cycle behavior and patterns of physical activity.

**Table 2:** Currently available measurement systems for sleep and circadian rhythms

**Measurement system**	**Key features**	**Proposed use**
Self-report	Prolonged reporting (for example, two weeks or more) of sleep timing, latency, quality and duration, daily mood, daytime physical activity	- Identification of insomnia, dysfunctional sleep and circadian-rhythms.
	Cross sectional questionnaires of sleep quality, excessive daytime sleepiness, fatigue, presence of sleep disordered breathing and circadian rhythms;	- Characterization of the sleep-wake cycle.
	Smart phone technologies now allow daily recording of sleep schedules and disturbances, as well as daytime physical activity patterns	- Assessment of treatment response.
Polysomnography	Laboratory or ambulatory monitoring of nocturnal sleep	- Differential diagnosis of various sleep disorders.
		- Characterization of sleep macro/microarchitecture.
Actigraphy	Indirect measure of the sleep-wake cycle especially convenient for multiday ambulatory assessment	- Identification of insomnia, dysfunctional sleep and circadian rhythms.
	At least one week of monitoring, (including weekdays and weekends)	- Characterization of the sleep-wake cycle.
	Key differentiation of patterns of sleep onset, offset, daytime activity and napping	- Assessment of treatment response.
	Some monitors can simultaneously record patterns of light exposure and estimate energy expenditure	
Melatonin assays	Dim light melatonin onset protocol in controlled laboratory settings or at home	- Characterization of circadian rhythms
		- Assessment of treatment response.
	From saliva, urine or blood	- Can support diagnosis of some circadian rhythm sleep disorders
Core body temperature monitoring	24-hour recording in controlled laboratory settings	- Characterization of circadian rhythms
		- Assessment of treatment response
	From ingested wireless probe or rectal probe	- Can support diagnosis of some circadian rhythm sleep disorders
Cortisol assays	24-hour recording	- Characterization of circadian rhythms
	From saliva, urine or blood	- Assessment of treatment response
Vigilance monitoring	24-hour recording in controlled laboratory settings	- Characterization of circadian rhythms
	- Psychomotor Vigilance Task (PVT)	- Assessment of treatment response
	- Wake EEG	
	- Multiple Sleep Latency test	
	The PVT or similar tasks are now available on smart phone applications	
Cardiovascular monitoring	Continuous or repeated measures of blood pressure and heart rate parameters across 24 hours in controlled laboratory settings	- Characterization of circadian rhythms
		- Assessment of treatment response

Within the research environment, the introduction of direct ecological monitoring systems [250,251] continues to provide important new insights into the daily patterns of physical activity, daytime resting, eating and other key social behaviors, alongside data related to subjective energy or mood levels. These types of studies have the capacity to reveal important longitudinal associations between daytime activity, sleep-wake cycle timing and mood and other psychological and neuropsychological variables.

#### Markedly delayed sleep phase in young people with depression

When the various sleep-wake technologies are applied in younger subjects in the early phases of major mood disorders, a quite different pattern of sleep and circadian rhythms emerges in comparison to young persons without mood disorders or middle-aged subjects with mood disorders. Our own studies highlight two key aspects in young persons with mood disorders, namely: (a) the predominance of delayed sleep phase (that is, not only going to bed later, but also rising later) [123] rather than the more characteristic pattern of phase advance that has traditionally been linked with more severe forms of depression in mid and later-life; and, (b) a late dim light melatonin onset (DLMO) compared to values classically found in young healthy persons [42,212]. This delayed pattern of the sleep-wake cycle and endogenous circadian rhythms is most marked in those with bipolar than unipolar phenotypes [123,212]. Importantly, longitudinal prospective studies are needed to identify sleep-wake or circadian predictors of mood disorders early in the course of illness. Notably, promising findings in youth have highlighted the predictive value of sleep problems and specific polysomnographic (PSG) markers for the future development of mood disorders [252-255] and the transition from unipolar to bipolar depression [256].

Such technologies can also be applied in older subjects with late-life depressive or early neurodegenerative disorders. Here, our data shows that greater nocturnal wakefulness as measured by actigraphic monitoring relates to neuropsychological dysfunction in late-life depression [43], mild cognitive impairment [257], and REM sleep behavior disorder in Parkinson’s disease [258]. However, in these cohorts, even cross-sectional self-report measures of sleep quality appear to be useful correlates of phenotypic features, including depression [259] and neuropsychological impairment [260].

Several PSG studies of persons with major depressive disorders have been conducted. Yet, PSG is costly and time consuming, often requires an adaptation night and requires time intensive scoring by sleep technicians. By contrast, self-report questionnaires, actigraphic recordings and sleep-wake cycle diaries are more accessible screening and measurement tools (Table 2). However, there is a clear need to link this new emphasis on extended actigraphic measurements of both sleep-wake cycle and circadian profiles with both PSG and more detailed measures of the full range of other indicators of circadian timing and synchrony (that is, body temperature, cortisol, melatonin and immune measures).

### Therapeutic approaches focused on correction of sleep-wake and circadian disturbance in major depression

While there is a long tradition of studies examining the effects of sleep-wake or circadian interventions in persons with major mood disorders (see Table 3), they do not figure prominently in major therapeutic guidelines for these common conditions [261,262]. By comparison with the size of large pharmaceutical trials focusing on modification of monoamine systems, and similarly psychological therapy trials focusing on cognitive, interpersonal and related behavioral factors, the evidence-base for sleep-wake and circadian interventions is only modest. Consequently, in major depression guidelines sleep and circadian-relevant interventions are referred to only as a general management strategy (for example, enhancing ‘sleep hygiene’ or promoting physical activity) or sleep and circadian related phenomena are viewed as possible presentations of alternative primary medical diagnosis (for example, sleep apnea).

**Table 3:** Therapeutic approaches for sleep and circadian disruptions in association with major depression

**Therapeutic approach**	**Target and rationale**
Psychoeducation	● Understanding sleep-wake and circadian regulation mechanisms and the processes through which sleep and circadian disturbances can be initiated and maintained
	● Linking changes in sleep quality, quantity and 24-hour sleep-wake cycles to onset and relapse of mood disorders
	● Promoting awareness of how daytime and nighttime behaviors and environmental factors influence sleep-wake and circadian rhythms (that is, sleep hygiene)
	N.B. These interventions are not considered to be efficient when used by themselves, but can be helpful in conjunction with other cognitive-behavioral interventions
Cognitive restructurig [286-288]	● Identifying and adjusting dysfunctional beliefs contributing to the maintenance of sleep difficulties
	● Understanding the influence of perceptions on sleep quality and daytime functioning
	● Establishing realistic expectations about sleep
	● Learning techniques to prevent evening/nighttime ruminations
Structured behavioral changes [143,266-272]	● Stimulus Control Therapy
	Aiming to reestablish positive associations between the bedroom and sleeping by: a) keeping the bedroom solely for sleep and sexual activities; and b) leaving the bedroom if awake for more than 15 minutes
	● Bed Restriction Therapy
	Using sleep pressure to enhance sleep consolidation by: a) limiting the sleep opportunity window to the habitual time spent asleep; and, b) increasing this window progressively as sleep efficiency (that is, ‘total sleep time’/‘time in bed’) improves
	● Rescheduling
	Progressive delay/advance of the sleep-wake and light–dark cycles
	● Regularization of wake-up times (emphasizing the importance of keeping the same wake-up times on weekends)
	● These techniques provide patients with simple therapeutic tools that can subsequently be used independently in case of relapse
Sleep deprivation [89,289-291]	● Can induce acute antidepressant effect
	● Can be used prior to sleep-wake rescheduling to facilitate sleep-wake phase shifting
	● May be useful to hasten and potentiate the response to phototherapy or cognitive-behavioral therapy
	N.B. Caution is warranted as sleep loss can trigger mania/hypomania episodes in patients with unipolar or bipolar depression
Intensive Sleep Retraining [292]	● While being monitored with polysomnography over a 25-hour protocol, patients are repeatedly given short sleep opportunities, each time being awoken shortly after achieving sleep (the progressive increase of sleep pressure is believed to facilitate multiple experiences of rapid sleep onset)
	● This novel conditioning technique may be especially promising for patients with depression and sleep/circadian disturbances because of the combined effects of acute partial sleep deprivation and subsequent improvement of sleep onset and other sleep parameters
	N.B. Caution is warranted as sleep loss can trigger mania or hypomania episodes
Social rhythms therapy for bipolar disorder [265]	● Integrated behavioral, interpersonal and psychoeducational therapy focusing on:
	- Regularizing daily activity rhythms (that is, eating, sleeping, leisure/work activities, social meetings)
	- Managing biological or psychosocial factors susceptible of dysregulating biological rhythms
	● Based on a model of bipolar disorder in which a genetic predisposition to circadian disturbances contributes to bipolar symptoms
Relaxation training [293-296]	● Techniques commonly used for insomnia include: progressive muscle relaxation, diaphragmatic breathing, autogenic training and imagery training
Phototherapy [271,273-278]	● Exposure to bright light (especially in the short blue to green wavelengths) has antidepressant and chronobiotic effects
	● To advances circadian rhythms:
	Morning exposure to bright light and evening exposure to dim light
	● To delay circadian rhythms:
	Evening exposure to bright light and morning exposure to dim light
	● Extended exposure to darkness can reduce manic symptoms in bipolar disorders
	● Actimeters with light sensors can be used to monitor adherence
Hypnotics/Sedatives	● In those with delayed circadian rhythms, can be used in the short-term to help realign the sleep phase to a regular schedule
Stimulant-wakefulness agents	● In those with daytime fatigue, low energy, reduced locomotor activity and daytime sleeping can been used to increase the wake period
	● Modafinil, a unique wakefulness agent, has been proposed as a treatment for bipolar disorder – including bipolar depression
Monoamine-based antidepressants [297,298]	● Often result in longer-term correction of sleep-wake cycle and circadian phase after recovery from depression – assumed via monoamine related mechanisms
	● Traditionally result in REM-sleep suppression and in the short-term may disturb sleep architecture
	● Those with more obvious noradrenergic properties have been used (with daytime or morning administration) to also promote daytime activity and arousal and help reduce subjective fatigue
	● Those with classical serotoninergic properties, when given at night, may increase arousal and wakefulness. While serotoninergic inputs to the SCN are expected to increase wakefulness, selective serotonin reuptake inhibitors (SSRIs) have not proved to be very useful in the management of more prolonged fatigue states compared with either nighttime sleep-promoting agents or daytime stimulants
Lithium [299-302]	● Inhibits *GSK-3*β, a kinase involved in the circadian regulation of the SCN
	● Modulates circadian rhythms (possibly by lengthening the circadian period or delaying endogenous rhythms)
	● Can enhance the therapeutic effects of combined sleep deprivation and phase advance in people with bipolar disorders
	● Known to decrease retinal sensitivity to light and could possibly influence melatonin’s sensitivity to light
Melatonin [303-305]	● Can advance sleep onset in those with delayed sleep phase syndrome
	● Could possibly improve the sleep-wake rhythm and prolong sleep in elderly people with advanced sleep phase syndrome (insufficient empirical data)
	● Reduce sleep onset latency and improve sleep efficiency (most consistent effects in elderly insomniac)
	N.B. Not recommended for children and adolescents under 18 years of age because of insufficient safety data (MIMS online)
Melatonin analogues [306-314]	● Direct effects on sleep onset with potential additional effects via other monoamine related mechanisms
	● Can reduce sleep onset latency and increase sleep duration in patients with insomnia
	● Could possibly be used to phase shift endogenous melatonin rhythms
	● Could possibly improve subjective sleep and increase sleep consolidation and SWS in patients with major depression
	● Can advance endogenous rhythms in older adults
	N.B. Not recommended for children and adolescents under 18 years of age because of insufficient safety data (MIMS online)

As most treatment guidelines (based on DSM-IV or International Classification of Diseases (ICD)-10) utilize a simple severity model (that is, mild/moderate/severe), for the clinical management of major depression it is not surprising that an approach focusing on a putative sleep-wake cycle or circadian pathophysiology is not central to treatment selection for people with major depressive disorders. Another difficulty is that relevant information is distributed across a broader literature notably in relation to bipolar disorder, primary insomnia, other primary sleep disorders, chronic fatigue, geriatric medicine and other areas of general medicine. One of the more attractive features of the various sleep and circadian-based antidepressant approaches (behavioral or pharmacological) available appears to be the relatively rapid onset of action. Given the much slower onset of action of conventional psychological or monoamine-based treatments, it is very surprising that this relative benefit is underemphasized in clinical practice [263,264].

#### Available circadian-based interventions

**Figure 4:**
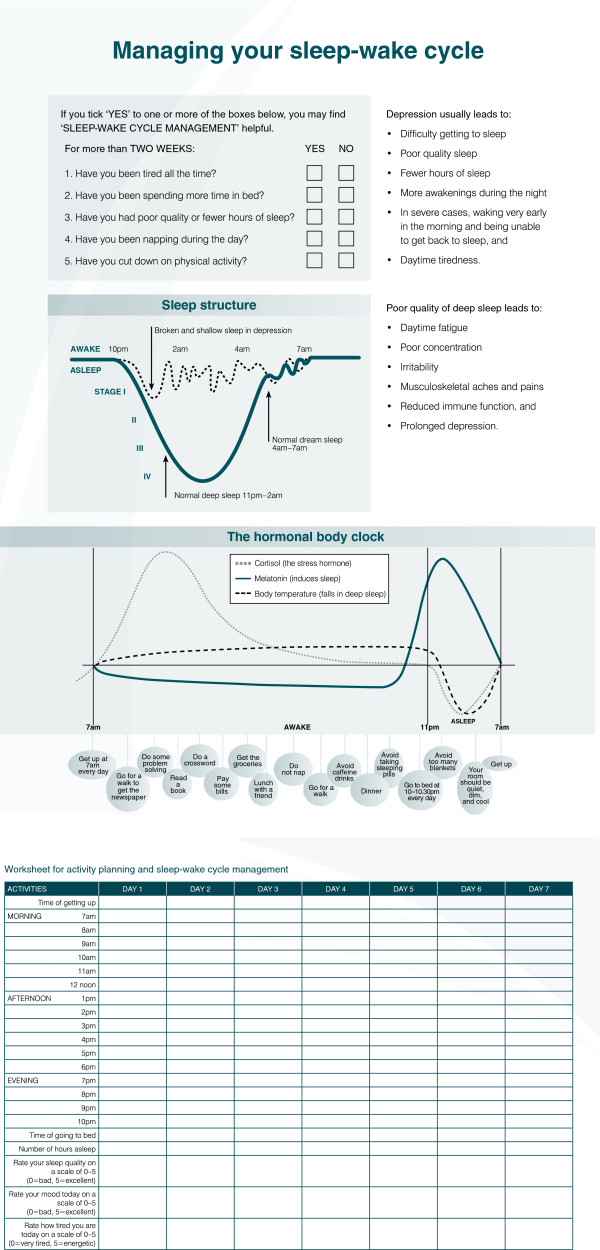
Psychoeducation and monitoring worksheet for patients with mood disorders and sleep-wake and circadian disturbance.

Despite these conceptual and organizational biases, it is possible to construct an extensive list of potentially therapeutic sleep-wake cycle and circadian interventions available for use in persons with major mood disorders (Table 3). Rather than providing separate information about nighttime/sleep *vs*. daytime/wake activity, this approach focuses on collating these perspectives within one framework focused on optimizing the 24-hour sleep-wake cycle. Additionally, it provides a more in-depth explanation of key aspects of circadian biology (for example, the importance of rising time and exposure to morning sunlight to suppress melatonin - see Figures 1 and 4). The tying of these factors with broader notions of social rhythms is also encouraged [265]. For those requiring more specific modifications of sleep-related phenomena, a range of key behavioral strategies is relevant [143,266-272]. Within this context, the use of specific forms of light therapy or daytime light exposure has received considerable attention [271,273-278].

#### Pharmacological approaches to sleep-wake and circadian systems

A wide range of pharmacotherapies has been utilized to manage sleep disturbance and related mood and cognitive phenomena [for a review, see [279]. Until recently, these have relied heavily on non-specific sedative strategies at night or stimulant-based approaches during daylight hours. These approaches, particularly when they are combined with appropriate other behavioral interventions, are likely to produce some short-term benefits. The place of traditional monoamine-based antidepressant strategies in the management of sleep and circadian related disorders remains controversial. While serotoninergic inputs to the SCN are consistent with the concept that these agents have the capacity to affect circadian patterns directly, the effects of these therapies on circadian parameters have not been studied extensively.

More often, the effects of monoamine-based therapies on key sleep parameters (sleep latency, REM latency, sleep architecture and suppression of REM sleep) have received attention [280-282]. The older tricyclic agents (TCAs) are still widely prescribed for their sedative properties and the extent to which more recent SSRI and selective norepinephrine reuptake inhibitor (SNRI) agents disturb sleep during the early phases of therapy (resulting in co-prescribing of hypnosedatives, TCAs or low dose antipsychotics) has been widely reported. Given the essentially activating inputs of serotonin to the SCN (see Figure 1), this effect is not surprising. It certainly raises questions about the optimal timing of administration of these compounds, which for convenience (or to minimize other side-effects) are often taken at night. The new question is whether any of the more recent melatonin-based products (particularly when taken at night) offer any specific benefits, from a sleep architecture, sleep-wake cycle, circadian or mood disorders perspective, compared with the conventional non-specific approaches.

#### Melatonin-based therapies

While melatonin has not traditionally been considered to be a viable antidepressant alone [283-285], the registration of agomelatine in the European Union and internationally (but not in the USA) as a treatment for major depression has again raised questions as to whether melatonin itself has significant antidepressant properties. What is more likely is that there are specific subgroups of those with major depressive disorders (and most obviously those with depression with chronic insomnia, circadian dysfunction, seasonal features or bipolar depression) who are likely to derive antidepressant benefits from melatonin, agomelatine or other melatonin-based analogues. Although agomelatine has been evaluated for short and longer-term efficacy by the European Medicines Agency, it has not been presented to the FDA for registration in the USA. Further, there has been considerable academic debate as to the relative efficacy of agomelatine compared with placebo or other available antidepressant agents [315-317]. Importantly, the differential effects of agomelatine as compared with SSRIs on sleep architecture has only recently been subject to investigation, with an initial study favoring the short-term benefits of agomelatine on key sleep parameters [318]. There is an ongoing need for further comparative studies. A very recent report suggests that another melatonin analogue, ramelteon, when taken adjunctively with other therapies may have benefits in preventing relapse into depression in patients with bipolar disorder [239].

#### Phase advance as a therapeutic strategy

What is most apparent is that therapies that result in phase advance in circadian rhythms are largely antidepressant in effect. The best studied examples are morning bright light therapies, partial sleep deprivation and enforced early morning rising [319-322]. Information from relevant pharmaceutical studies indicate that SSRI drugs induce a phase advance in the firing of SCN neurons in culture and that agomelatine induces a phase advance in endogenous circadian rhythms [322,323]. These phase-advancing effects of monoamine and melatonin-acting agents are contrasted with those of lithium, which can induce a phase delay [290,324-326]. However, in clinical studies lithium has its principal effect on mania (and prevention of manic relapse) rather than acute depressive states. Other novel pharmacological agents are under investigation for their capacity to induce a phase advance or restore normal circadian patterns (acting via effects on other regulatory proteins, such as *CK1-delta*). However, it is not yet known whether such agents have antidepressant or other mood stabilizing properties (See [58]).

The development of novel therapeutic strategies for circadian-based mood disorders (and other closely-related neuropsychiatric syndromes) is now likely to focus on behavioral or pharmacological strategies that: (i) phase advance circadian timing, as this is the strongest correlate of an antidepressant response. This may be achieved by focusing on SCN molecular targets to alter period length or by making greater use of behavioral or pharmacological approaches that entrain or enhance melatonin secretion; (ii) entrain rhythms more robustly in those clinical situations in which there is strong evidence of only weak linkage to the day-night cycle or evidence of chaotic or frequently-changing patterns. In clinical psychiatry, the depressive and mixed mood phases of bipolar disorder are likely to be the main targets. These may include strategies akin to lithium and may be associated with phase delay or phase advance in timing. Current melatonin-based strategies may also be central to this approach; and (iii) restore normal internal synchrony of key behavioral, neurohormonal, metabolic, autonomic and immunological systems. This may be achieved by a variety of existing mechanisms (for example, behavioral and pharmacological approaches to enhance melatonin release) or novel mechanisms that target the molecular mechanisms of the SCN, melatonin release by the pineal gland or factors that modulate other key hypocretin/orexin or leptin-associated mechanisms.

### Planning a mood disorder intervention based on sleep and circadian therapies

In those subjects with mood disorders who have been identified as presenting key features consistent with underlying perturbations of sleep-wake cycle or circadian systems, it is possible to set out a therapeutic program over a six- to eight-week period (Table 4). The key features are strong reliance on self-monitoring, highly structured planning of waking, activity, sunlight (or adjunctive light) exposure and sleep schedules. Strong educational elements about the underlying neurobiology and frequent reviews of difficulties encountered, as well as careful monitoring of the mood disorder and associated risks are essential (see Figure 4). In this process, decisions about adjunctive specific pharmacotherapy for either the circadian or depression elements are delayed until at least four weeks, although the use of melatonin may be considered after two weeks.

**Table 4:** Planning an individualized sleep and circadian intervention

**A. Initial clinical assessment and enrollment to two weeks of systematic assessment**	
1. Clinical assessment of depression	Assessment for key features indicative of circadian-dependent mood disorders including:
	● Positive family history of mania or circadian rhythm sleep disorders
	● Diurnal or seasonal sensitivity
	● Easy destabilization by changes in time-zones or changes in regular sleep pattern
	● Non-restorative sleep
	● Daytime fatigue
	● Difficulty falling asleep
	● Late morning rising or waking up early in the morning
	● Oversleeping
	● Overeating or weight gain
	● Screen for other sleep disorders, such as restless legs syndrome or sleep apnea
2. Evaluation of key sleep and circadian phenotypes	Self-report/self-monitoring over two-weeks (see Figure 4) using smart phone or paper-pencil, particularly focusing on:
	● Chronotypes on morningness-eveningness scales
	● work/schooldays and weekend schedules
	● Duration of sleep
	● Waking from sleep
	● Pre-sleep hyperarousal symptoms
	● Night sweats – raised temperature during sleep
	● Timing and level of daytime physical activity
	● Atypical circadian mood variations
	Objective measures including:
	● Two-weeks of continuous actigraphy/sleep diaries
	● Dim light melatonin onset assays
	B) Information and treatment planning sessions
1. Psychoeducation with regards to the human sleep and circadian systems	Key elements include:
	● Explanation of the biology of the human clock
	● Illustration of the normal 24-hour cycle in sleep and activity and synchronization with hormonal, immune, body temperature and other key physiological elements
	● Emphasis on setting the clock through morning rising, appropriately timed light exposure, regularity of activity cycles, daytime physical activity, bedtime schedules and nighttime practices
	● Linking to eating behavior and risks to obesity and metabolic function
2. Set specific behavioral elements	Key decisions include:
	● Set sleep offset time (or schedule for gradual phase advance/delay relative to current waking time) with special care to avoid sleep loss induced mania/hypomania episode in people at risk for bipolar disorder
	● Set daily activity schedules
	● Emphasize morning light exposure (natural or through specific devices with special care to avoid bright light induced mania/hypomania in people at risk for bipolar disorder)
	● Discuss regular sleep onset time expectations
	● Set sleeping conditions relative to light exposure and temperature
3. Introduce self-report or objective measurement techniques for this period	Key elements include (see Figure 4):
	● Daily monitoring of actual sleep onset /offset, sleep duration and sleep quality
	● Continuous recording of actual daytime physical activity
	● Daily mood and fatigue monitoring ● Monitoring of substance use and eating behavior ● Monitoring of other behaviors that could adversely affect sleep including excessive or poorly timed napping
C) Review progress at two weeks	
	Key elements are:
	● Adherence to sleep offset time, light exposure and degree of actual physical activity
	● Evaluate changes in daily mood, fatigue, sleep quality
D) If inadequate clinical progress:	
	Consider:
	● Adherence and planning issues
	● Adjunctive strategies to be considered:
	- Earlier/later or augmented light exposure
	- Melatonin supplementation with careful planning of ingestion time
E) Review progress at four weeks	
If inadequate clinical progress:	Consider:
	● Adherence and planning issues
	● Adjunctive strategies to be considered:
	- Melatonin-based antidepressant strategies
	- Other conventional antidepressant strategies
	- Alternative daytime stimulant or nighttime sedation strategies
F) Review progress at six- to eight-weeks	

A key consideration in such programs is the age of the patients and the direction and degree of phase shift. In younger subjects, there is a normal developmental change in sleep and circadian rhythms with a shift towards phase delay throughout the late adolescent period. Hence, phase advancing strategies in this group need to be cognizant of a return to normal developmental patterns. For older subjects, the converse may be in operation with a marked shift towards earlier sleep onset and sleep offset times. In relation to both sets of arrangements, it is as important to focus on the degree and timing of daytime activity and other related behaviors (for example, controlling excessive daytime napping) as it is to focus on nighttime behavior.

#### Limitations of circadian-based assessments and therapeutics

At this time, the style of prolonged actigraphy-based sleep-wake cycle measurements - that are essential for personalizing treatment regimens - are not in common clinical use. Hence, there is a need to introduce them more widely and for their use to be augmented by appropriate prolonged reports of mood, behavior and sleep quality (that is, ‘mood, sleep and behavior diaries’ – see Figure 4). Importantly, such measures are only indirect indicators of underlying circadian biology. That is, they cannot be used to confirm the presence of internal desynchrony, as this requires simultaneous monitoring over at least a 24-hour period of various endogenous measures, such as melatonin, cortisol or core body temperature. However, as body temperature recordings and other physiological measures become more widely available, these may also become more consistently used, especially if empirical studies continue to refine our understanding of the evolution of these markers across the various stages of affective illnesses.

From a therapeutic perspective, the direct or indirect effects of many available psychotropic compounds on circadian biology have not yet been clarified. Although the availability of melatonin and melatonin-based agonists has already had an impact in the insomnia field, its direct relevance to those with primary mood (including both unipolar and bipolar) disorders is still unclear. With the emergence of hypocretin/orexin antagonists, it is likely that further complexities will become apparent.

The imposition of rigid behavioral or timing schedules on different groups of patients is clinically challenging. In general terms, this is particularly so for younger subjects who experience a normal adolescent period of phase delay (and may also need to fit with school or other educational schedules) and older subjects who may be experiencing significant phase advance. Additionally, those with different underlying chronotypes (‘morningness’ vs. ‘eveningness’) will vary in their capacity to incorporate major changes in daytime scheduling into their lifestyle.

## Summary

There is an urgent need in clinical psychiatry to advance more personalized approaches to syndrome description and treatment selection. In our view, this goal is now within reach, at least for that subpopulation of people with mood disorders who have clear evidence of disturbed circadian physiology. Current estimates among adult populations (based on the bipolar spectrum concept) indicate that this may be in the order of 40% of middle-aged patients [327]. This departure from traditional antidepressant strategies has been possible due to four factors: (i) major advances in the basic neurosciences of sleep and the related circadian systems, with great detailing of the molecular machinery of the circadian clock and the processes by which it both responds to external cues and regulates internal physiology; (ii) developmental studies of sleep-wake cycle and circadian parameters of people (teenagers, young adults and older persons) close to the onset of their major depression; (iii) use of more objective and specific measurement assays and longer-term monitoring systems for circadian dysfunction in humans; and, (iv) development of new and more specific behavioral (incorporating education, sleep rescheduling, light exposure and daytime physical activity) and pharmacological (largely melatonin-based) therapies for treatment of mood disorders. A much greater focus on circadian-based major depressive disorders has the potential to deliver clinical psychiatry what it has always desired – a diagnostic grouping linked with strong objective measures of illness state and a basis for more rational treatment selection and monitoring of response to targeted behavioral or pharmacological interventions.

## Abbreviations

ACTH: Adrenocorticotropic hormone; CLOCK: Circadian Locomotor Output Cycles Kaput; CK: Casein kinase; CNS: Central nervous system; GSK-3β: Glycogen synthase kinase 3 beta; DLMO: Dim light melatonin onset; DSM: Diagnostic and Statistical Manual of Mental Disorders; HPA: Hypothalamus-pituitary-adrenal; ICD: International Classification of Diseases; PSG: Polysomnography; PVN: Paraventricular nucleus; PVT: Psychomotor Vigilance Test; REM: Rapid eye movement; SCN: Suprachiasmatic nucleus; SNRI: Selective norepinephrine reuptake inhibitor; SSRI: Selective serotonin reuptake inhibitor; SWS: Slow wave (or deep) sleep; TCA: Tricyclic antidepressant; TSH: Thyroid stimulating hormone

## Competing interests

IBH has led a range of community-based and pharmaceutical industry-supported depression awareness and education and training programs. He has led depression and other mental health research projects that have been supported by a variety of pharmaceutical partners. Current investigator-initiated studies are supported by Servier and Pfizer. He has received honoraria for his contributions to professional educational seminars related to depression, youth mental health and circadian rhythms research. EMS has received honoraria for educational seminars related to the clinical management of depressive disorders supported by Servier and Eli-Lilly pharmaceuticals. She has participated in a national advisory board for the antidepressant compound Pristiq, manufactured by Pfizer. DFH has received honoraria for educational seminars from Janssen-Cilag.

## Authors’ contributions

All authors discussed the evidence and contributed to the writing of this manuscript, primarily through a significant discourse undertaken over the past five years. All authors read and approved the final manuscript.

## Pre-publication history

The pre-publication history for this paper can be accessed here:

http://www.biomedcentral.com/1741-7015/11/79/prepub

## References

[B1] Horowitz AV Wakefield JC The Loss of Sadness: How Psychiatry Transformed Normal Sorrow into Depressive Disorder 2007 New York: Oxford University Press 10.1176/appi.ajp.2007.07081263 22688233

[B2] Shorter E Before Prozac: The Troubled History of Mood Disorders in Psychiatry 2009 New York: Oxford University Press

[B3] DSM-5: the future of psychiatric diagnosis http://www.dsm5.org/Pages/Default.aspx

[B4] Diagnosing the D.S.M http://www.nytimes.com/2012/05/12/opinion/break-up-the-psychiatric-monopoly.html

[B5] Psychiatry’s bible, the DSM, is doing more harm than good http://articles.washingtonpost.com/2012-04-27/opinions/35450916_1_bipolar-disorder-psychiatric-ward-psychiatric-drugs

[B6] Hyman SE Can neuroscience be integrated into the DSM-V? Nat Rev Neurosci 2007 8 725 732 17704814 10.1038/nrn2218

[B7] Miller G Is pharma running out of brainy ideas? Science 2010 329 502 504 20671165 10.1126/science.329.5991.502

[B8] Lopez AD Mathers CD Ezzati M Jamison DT Murray CJL Global and regional burden of disease and risk factors, 2001: systematic analysis of population health data Lancet 2006 367 1747 1757 16731270 10.1016/S0140-6736(06)68770-9

[B9] Gustavsson A Svensson M Jacobi F Allgulander C Alonso J Beghi E Dodel R Ekman M Faravelli C Fratiglioni L Gannon B Jones DH Jennum P Jordanova A Jonsson L Karampampa K Knapp M Kobelt G Kurth T Lieb R Linde M Ljungcrantz C Maercker A Melin B Moscarelli M Musayev A Norwood F Preisig M Pugliatti M Rehm J Cost of disorders of the brain in Europe 2010 Eur Neuropsychopharmacol 2011 21 718 779 21924589 10.1016/j.euroneuro.2011.08.008

[B10] Bloom DE Cafiero ET Jané-Llopis E Abrahams-Gessel S Bloom LR Fathima S Feigl AB Gaziano T Mowafi M Pandya A Prettner K Rosenberg L Seligman B Stein AZ Weinstein C The Global Economic Burden of Non-Communicable Diseases 2011 Geneva: World Economic Forum Available at: http://www3.weforum.org/docs/WEF_Harvard_HE_GlobalEconomicBurdenNonCommunicableDiseases_2011.pdf

[B11] Insel TR The arrival of preemptive psychiatry Early Interv Psychiatry 2007 1 5 6 21352102 10.1111/j.1751-7893.2007.00017.x

[B12] Insel TR Translating scientific opportunity into public health impact: a strategic plan for research on mental illness Arch Gen Psychiatry 2009 66 128 133 19188534 10.1001/archgenpsychiatry.2008.540

[B13] Hickie IB Scott EM Hermens DF Naismith SL Guastella AJ Kaur M Sidis A Whitwell B Glozier N Pantelis C Wood SJ McGorry PD Applying a clinical staging framework in young people who present with admixtures of anxious, depressive or psychotic symptoms Early Interv Psychiatry 2013 7 31 43 22672533 10.1111/j.1751-7893.2012.00366.x

[B14] Scott EM Hermens DF Glozier N Naismith SL Guastella AJ Hickie IB Targeted primary care-based mental health services for young Australians Med J Aust 2012 196 136 140 22304610 10.5694/mja11.10481

[B15] McGorry PD Yung AR Pantelis C Hickie IB A clinical trials agenda for testing interventions in earlier stages of psychotic disorders Med J Aust 2009 190 S33 S36 19220171 10.5694/j.1326-5377.2009.tb02372.x

[B16] Hickie IB Naismith SL Norrie LM Scott EM Managing depression across the life cycle: new strategies for clinicians and their patients Intern Med J 2009 39 720 727 19912400 10.1111/j.1445-5994.2009.02016.x

[B17] Hickie IB Naismith SL Ward PB Little CL Pearson M Scott EM Mitchell P Wilhelm K Parker G Psychomotor slowing in older patients with major depression: relationships with blood flow in the caudate nucleus and white matter lesions Psychiatry Res 2007 155 211 220 17574392 10.1016/j.pscychresns.2007.01.006

[B18] Hickie I Naismith S Ward PB Turner K Scott E Mitchell P Wilhelm K Parker G Reduced hippocampal volumes and memory loss in patients with early- and late-onset depression Br J Psychiatry 2005 186 197 202 15738499 10.1192/bjp.186.3.197

[B19] Naismith SL Norrie LM Mowszowski L Hickie IB The neurobiology of depression in later-life: clinical, neuropsychological, neuroimaging and pathophysiological features Prog Neurobiol 2012 98 99 143 22609700 10.1016/j.pneurobio.2012.05.009

[B20] Carroll BJ Dexamethasone suppression test: a review of contemporary confusion J Clin Psychiatry 1985 46 13 24 3881417

[B21] Carroll BJ Informed use of the dexamethasone suppression test J Clin Psychiatry 1986 47 10 12 3941065

[B22] Wray NR Pergadia ML Blackwood DH Penninx BW Gordon SD Nyholt DR Ripke S MacIntyre DJ McGhee KA Maclean AW Smit JH Hottenga JJ Willemsen G Middeldorp CM de Geus EJ Lewis CM McGuffin P Hickie IB van den Oord EJ Liu JZ Macgregor S McEvoy BP Byrne EM Medland SE Statham DJ Henders AK Heath AC Montgomery GW Martin NG Boomsma DI Genome-wide association study of major depressive disorder: new results, meta-analysis, and lessons learned Mol Psychiatry 2010 17 36 48 21042317 10.1038/mp.2010.109 PMC3252611

[B23] Thase ME Larsen KG Kennedy SH Assessing the 'true' effect of active antidepressant therapy v. placebo in major depressive disorder: use of a mixture model Br J Psychiatry 2011 199 501 507 22130749 10.1192/bjp.bp.111.093336

[B24] Turner EH Matthews AM Linardatos E Tell RA Rosenthal R Selective publication of antidepressant trials and its influence on apparent efficacy N Engl J Med 2008 358 252 260 18199864 10.1056/NEJMsa065779

[B25] Fournier JC DeRubeis RJ Hollon SD Dimidjian S Amsterdam JD Shelton RC Fawcett J Antidepressant drug effects and depression severity: a patient-level meta-analysis JAMA 2010 303 47 53 20051569 10.1001/jama.2009.1943 PMC3712503

[B26] Simon GE How can we know whether antidepressants increase suicide risk? Am J Psychiatry 2006 163 1861 1863 17074930 10.1176/ajp.2006.163.11.1861

[B27] Yung AR McGorry PD The initial prodrome in psychosis: descriptive and qualitative aspects Aust N Z J Psychiatry 1996 30 587 599 8902166 10.3109/00048679609062654

[B28] Yung AR Stanford C Cosgrave E Killackey E Phillips L Nelson B McGorry PD Testing the Ultra High Risk (prodromal) criteria for the prediction of psychosis in a clinical sample of young people Schizophr Res 2006 84 57 66 16630707 10.1016/j.schres.2006.03.014

[B29] Amminger GP Leicester S Yung AR Phillips LJ Berger GE Francey SM Yuen HP McGorry PD Early-onset of symptoms predicts conversion to non-affective psychosis in ultra-high risk individuals Schizophr Res 2006 84 67 76 16677803 10.1016/j.schres.2006.02.018

[B30] Bromet E Andrade L Hwang I Sampson N Alonso J de Girolamo G de Graaf R Demyttenaere K Hu C Iwata N Karam A Kaur J Kostyuchenko S Lepine J-P Levinson D Matschinger H Mora M Browne M Posada-Villa J Viana M Williams D Kessler R Cross-national epidemiology of DSM-IV major depressive episode BMC Med 2011 9 90 21791035 10.1186/1741-7015-9-90 PMC3163615

[B31] Scott EM Hermens DF Naismith SL Guastella AJ De Regt T White D Lagopoulos J Hickie IB Distinguishing young people with emerging bipolar disorders from those with unipolar depression J Affect Disord 2013 144 208 215 22877963 10.1016/j.jad.2012.06.031

[B32] Hickie IB Luscombe GM Davenport TA Burns JM Highet NJ Perspectives of young people on depression: awareness, experiences, attitudes and treatment preferences Early Interv Psychiatry 2007 1 333 339 21352121 10.1111/j.1751-7893.2007.00042.x

[B33] Hamilton BA Naismith SL Scott EM Purcell S Hickie IB Disability is already pronounced in young people with early stages of affective disorders: Data from an early intervention service J Affect Disord 2011 131 84 91 21112640 10.1016/j.jad.2010.10.052

[B34] Hickie IB Rogers NL Novel melatonin-based therapies: potential advances in the treatment of major depression Lancet 2011 378 621 631 21596429 10.1016/S0140-6736(11)60095-0

[B35] Germain A Kupfer DJ Circadian rhythm disturbances in depression Hum Psychopharmacol Clin Exp 2008 23 571 585 10.1002/hup.964 PMC2612129 18680211

[B36] Pandi-Perumal SR Moscovitch A Srinivasan V Spence DW Cardinali DP Brown GM Bidirectional communication between sleep and circadian rhythms and its implications for depression: lessons from agomelatine Prog Neurobiol 2009 88 264 271 19454302 10.1016/j.pneurobio.2009.04.007

[B37] Boivin DB Influence of sleep-wake and circadian rhythm disturbances in psychiatric disorders J Psychiatry Neurosci 2000 25 446 458 11109296 PMC1408010

[B38] Van den Hoofdakker RH Chronobiological theories of nonseasonal affective disorders and their implications for treatment J Biol Rhythms 1994 9 157 183 7873775 10.1177/074873049400900206

[B39] Glozier N Martiniuk A Patton G Ivers R Li Q Hickie I Senserrick T Woodward M Norton R Stevenson M Short sleep duration in prevalent and persistent psychological distress in young adults: the DRIVE study Sleep 2010 33 1139 1145 20857859 10.1093/sleep/33.9.1139 PMC2938854

[B40] Boivin DB Czeisler CA Dijk DJ Duffy JF Folkard S Minors DS Totterdell P Waterhouse JM Complex interaction of the sleep-wake cycle and circadian phase modulates mood in healthy subjects Arch Gen Psychiatry 1997 54 145 152 9040282 10.1001/archpsyc.1997.01830140055010

[B41] Surridge-David M MacLean A Coulter ME Knowles JB Mood change following an acute delay of sleep Psychiatry Res 1987 22 149 158 Erratum: *Psychiatry Res* 1988, 24:121 3685222 10.1016/0165-1781(87)90102-8

[B42] Naismith SL Hermens DF Ip TK Bolitho S Scott E Rogers NL Hickie IB Circadian profiles in young people during the early stages of affective disorder Transl Psychiatry 2012 2 e123 22832967 10.1038/tp.2012.47 PMC3365266

[B43] Naismith SL Rogers NL Lewis SJ Terpening Z Ip T Diamond K Norrie L Hickie IB Sleep disturbance relates to neuropsychological functioning in late-life depression J Affect Disord 2011 132 139 145 21435728 10.1016/j.jad.2011.02.027

[B44] Storch EA Murphy TK Lack CW Geffken GR Jacob ML Goodman WK Sleep-related problems in pediatric obsessive-compulsive disorder J Anxiety Dis 2008 22 877 885 10.1016/j.janxdis.2007.09.003 PMC2413417 17951025

[B45] Robillard R Lambert T Whitwell BG Ip TK Hickie IB Rogers NL Sleep-wake patterns and mood disturbances in patients with psychotic disorders: a controlled study Proceedings of the 4th International Congress of the Association of Sleep Medicine (WASM) & 5th Conference of the Canadian Sleep Society (CSS) September 2011 10 14 *Quebec City*. *Sleep Med* 2011, 12:S53

[B46] Mohawk JA Green CB Takahashi JS Central and peripheral circadian clocks in mammals Annu Rev Neurosci 2012 35 445 462 22483041 10.1146/annurev-neuro-060909-153128 PMC3710582

[B47] Albrecht U Timing to perfection: the biology of central and peripheral circadian clocks Neuron 2012 74 246 260 22542179 10.1016/j.neuron.2012.04.006

[B48] Welsh DK Takahashi JS Kay SA Suprachiasmatic nucleus: cell autonomy and network properties Annu Rev Physiol 2010 72 551 577 20148688 10.1146/annurev-physiol-021909-135919 PMC3758475

[B49] Lowrey PL Takahashi JS Genetics of circadian rhythms in mammalian model organisms Adv Genet 2011 74 175 230 21924978 10.1016/B978-0-12-387690-4.00006-4 PMC3709251

[B50] Koike N Yoo SH Huang HC Kumar V Lee C Kim TK Takahashi JS Transcriptional architecture and chromatin landscape of the core circadian clock in mammals Science 2012 338 349 354 22936566 10.1126/science.1226339 PMC3694775

[B51] Panda S Hogenesch JB It's all in the timing: many clocks, many outputs J Biol Rhythms 2004 19 374 387 15534318 10.1177/0748730404269008

[B52] Takahashi JS Shimomura K Kumar V Searching for genes underlying behavior: lessons from circadian rhythms Science 2008 322 909 912 18988844 10.1126/science.1158822 PMC3744585

[B53] Chen Z Yoo SH Takahashi JS Small molecule modifiers of circadian clocks Cell Mol Life Sci 2012 Epub ahead of print 10.1007/s00018-012-1207-y PMC3760145 23161063

[B54] Etchegaray JP Yu EA Indic P Dallmann R Weaver DR Casein kinase 1 delta (CK1delta) regulates period length of the mouse suprachiasmatic circadian clock in vitro PLoS One 2010 5 e10303 20421981 10.1371/journal.pone.0010303 PMC2858662

[B55] Etchegaray JP Machida KK Noton E Constance CM Dallmann R Di Napoli MN DeBruyne JP Lambert CM Yu EA Reppert SM Weaver DR Casein kinase 1 delta regulates the pace of the mammalian circadian clock Mol Cell Biol 2009 29 3853 3866 19414593 10.1128/MCB.00338-09 PMC2704743

[B56] Xu Y Padiath QS Shapiro RE Jones CR Wu SC Saigoh N Saigoh K Ptácek LJ Fu YH Functional consequences of a CKIdelta mutation causing familial advanced sleep phase syndrome Nature 2005 434 640 644 15800623 10.1038/nature03453

[B57] Vatine G Vallone D Gothilf Y Foulkes NS It’s time to swim! Zebrafish and the circadian clock FEBS Lett 2011 585 1485 1494 21486566 10.1016/j.febslet.2011.04.007

[B58] McClung CA Circadian rhythms and mood regulation: insights from pre-clinical models Eur Neuropsychopharmacol 2011 21 Supplement 4 S683 S693 21835596 10.1016/j.euroneuro.2011.07.008 PMC3179573

[B59] Cade JF Lithium salts in the treatment of psychotic excitement Med J Aust 1949 2 349 352 18142718 10.1080/j.1440-1614.1999.06241.x

[B60] Hirota T Lewis WG Liu AC Lee JW Schultz PG Kay SA A chemical biology approach reveals period shortening of the mammalian circadian clock by specific inhibition of GSK-3 beta Proc Natl Acad Sci U S A 2008 105 20746 20751 19104043 10.1073/pnas.0811410106 PMC2606900

[B61] Lachman HM Pedrosa E Petruolo OA Cockerham M Papolos A Novak T Papolos DF Stopkova P Increase in GSK3β gene copy number variation in bipolar disorder Am J Med Genet B Neuropsychiatr Genet 2007 144B 259 265 17357145 10.1002/ajmg.b.30498

[B62] Kozikowski AP Gunosewoyo H Guo S Gaisina IN Walter RL Ketcherside A McClung CA Mesecar AD Caldarone B Identification of a glycogen synthase kinase-3beta inhibitor that attenuates hyperactivity in CLOCK mutant mice Chem Med Chem 2011 6 1593 1602 21732538 10.1002/cmdc.201100188 PMC3428230

[B63] Arey R McClung CA An inhibitor of casein kinase 1 epsilon/delta partially normalizes the manic-like behaviors of the ClockDelta19 mouse Behav Pharmacol 2012 23 392 396 22743604 10.1097/FBP.0b013e32835651fd PMC3673712

[B64] Isojima Y Nakajima M Ukai H Fujishima H Yamada RG Masumoto KH Kiuchi R Ishida M Ukai-Tadenuma M Minami Y Kito R Nakao K Kishimoto W Yoo SH Shimomura K Takao T Takano A Kojima T Nagai K Sakaki Y Takahashi JS Ueda HR CKIepsilon/delta-dependent phosphorylation is a temperature-insensitive, period-determining process in the mammalian circadian clock Proc Natl Acad Sci U S A 2009 106 15744 15749 19805222 10.1073/pnas.0908733106 PMC2736905

[B65] Mendoza J Clesse D Pévet P Challet E Food-reward signalling in the suprachiasmatic clock J Neurochem 2010 112 1489 1499 20067576 10.1111/j.1471-4159.2010.06570.x

[B66] Mendoza J Circadian clocks: setting time by food J Neuroendocrinol 2007 19 127 137 17214875 10.1111/j.1365-2826.2006.01510.x

[B67] Van Gelder RN How the clock sees the light Nat Neurosci 2008 11 628 630 18506138 10.1038/nn0608-628

[B68] Guido ME Garbarino-Pico E Contin MA Valdez DJ Nieto PS Verra DM Acosta-Rodriguez VA de Zavalía N Rosenstein RE Inner retinal circadian clocks and non-visual photoreceptors: novel players in the circadian system Prog Neurobiol 2010 92 484 504 20736045 10.1016/j.pneurobio.2010.08.005

[B69] Cermakian N Sassone-Corsi P Environmental stimulus perception and control of circadian clocks Curr Opin Neurobiol 2002 12 359 365 12139981 10.1016/s0959-4388(02)00347-1

[B70] Hidalgo MP Caumo W Posser M Coccaro SB Camozzato AL Chaves ML Relationship between depressive mood and chronotype in healthy subjects Psychiatry Clin Neurosci 2009 63 283 290 19566758 10.1111/j.1440-1819.2009.01965.x

[B71] Drennan MD Klauber MR Kripke DF Goyette LM The effects of depression and age on the Horne-Ostberg morningness-eveningness score J Affect Disord 1991 23 93 98 1753041 10.1016/0165-0327(91)90096-b

[B72] Hakkarainen R Johansson C Kieseppa T Partonen T Koskenvuo M Kaprio J Lonnqvist J Seasonal changes, sleep length and circadian preference among twins with bipolar disorder BMC Psychiatry 2003 3 6 12795811 10.1186/1471-244X-3-6 PMC165438

[B73] Dzirasa K Coque L Sidor MM Kumar S Dancy EA Takahashi JS McClung CA Nicolelis MA Lithium ameliorates nucleus accumbens phase-signaling dysfunction in a genetic mouse model of mania J Neurosci 2010 30 16314 16323 21123577 10.1523/JNEUROSCI.4289-10.2010 PMC3165036

[B74] Roybal K Theobold D Graham A DiNieri JA Russo SJ Krishnan V Chakravarty S Peevey J Oehrlein N Birnbaum S Vitaterna MH Orsulak P Takahashi JS Nestler EJ Carlezon WA Jr McClung CA Mania-like behavior induced by disruption of CLOCK Proc Natl Acad Sci U S A 2007 104 6406 6411 17379666 10.1073/pnas.0609625104 PMC1851061

[B75] McClung CA Sidiropoulou K Vitaterna M Takahashi JS White FJ Cooper DC Nestler EJ Regulation of dopaminergic transmission and cocaine reward by the clock gene Proc Natl Acad Sci U S A 2005 102 9377 9381 15967985 10.1073/pnas.0503584102 PMC1166621

[B76] Spencer S Torres-Altoro MI Falcon E Arey R Marvin M Goldberg M Bibb JA McClung CA A mutation in CLOCK leads to altered dopamine receptor function J Neurochem 2012 123 124 134 22757753 10.1111/j.1471-4159.2012.07857.x PMC3438377

[B77] Spencer S Falcon E Kumar J Krishnan V Mukherjee S Birnbaum SG McClung CA Circadian genes period 1 and period 2 in the nucleus accumbens regulate anxiety-related behavior Eur J Neurosci 2013 37 242 250 23039899 10.1111/ejn.12010 PMC3711746

[B78] Mongrain V Cermakian N Clock genes in health and disease J Appl Biomed 2009 7 15 33

[B79] Takahashi JS Hong HK Ko CH McDearmon EL The genetics of mammalian circadian order and disorder: implications for physiology and disease Nat Rev Genet 2008 9 764 775 18802415 10.1038/nrg2430 PMC3758473

[B80] Hardeland R Cardinali DP Srinivasan V Spence DW Brown GM Pandi-Perumal SR Melatonin - a pleiotropic, orchestrating regulator molecule Prog Neurobiol 2011 93 350 384 21193011 10.1016/j.pneurobio.2010.12.004

[B81] Shimomura K Lowrey PL Vitaterna MH Buhr ED Kumar V Hanna P Omura C Izumo M Low SS Barrett RK LaRue SI Green CB Takahashi JS Genetic suppression of the circadian clock mutation by the melatonin biosynthesis pathway Proc Natl Acad Sci U S A 2010 107 8399 8403 20404168 10.1073/pnas.1004368107 PMC2889547

[B82] Bertolucci C Cavallari N Colognesi I Aguzzi J Chen Z Caruso P Foa A Tosini G Bernardi F Pinotti M Evidence for an overlapping role of CLOCK and NPAS2 transcription factors in liver circadian oscillators Mol Cell Biol 2008 28 3070 3075 18316400 10.1128/MCB.01931-07 PMC2293078

[B83] Stokkan KA Yamazaki S Tei H Sakaki Y Menaker M Entrainment of the circadian clock in the liver by feeding Science 2001 291 490 493 11161204 10.1126/science.291.5503.490

[B84] Storch KF Lipan O Leykin I Viswanathan N Davis FC Wong WH Weitz CJ Extensive and divergent circadian gene expression in liver and heart Nature 2002 417 78 83 11967526 10.1038/nature744

[B85] Muhlbauer E Wolgast S Finckh U Peschke D Peschke E Indication of circadian oscillations in the rat pancreas FEBS Lett 2004 564 91 96 15094047 10.1016/S0014-5793(04)00322-9

[B86] Sadacca LA Lamia KA DeLemos AS Blum B Weitz CJ An intrinsic circadian clock of the pancreas is required for normal insulin release and glucose homeostasis in mice Diabetologia 2011 54 120 124 20890745 10.1007/s00125-010-1920-8 PMC2995870

[B87] Bass J Takahashi JS Circadian integration of metabolism and energetics Science 2010 330 1349 1354 21127246 10.1126/science.1195027 PMC3756146

[B88] Solt LA Wang Y Banerjee S Hughes T Kojetin DJ Lundasen T Shin Y Liu J Cameron MD Noel R Yoo SH Takahashi JS Butler AA Kamenecka TM Burris TP Regulation of circadian behaviour and metabolism by synthetic REV-ERB agonists Nature 2012 485 62 68 22460951 10.1038/nature11030 PMC3343186

[B89] Borbely AA Wirz-Justice A Sleep, sleep deprivation and depression. A hypothesis derived from a model of sleep regulation Hum Neurobiol 1982 1 205 210 7185793

[B90] Adamantidis A de Lecea L Sleep and metabolism: shared circuits, new connections Trends Endocrinol Metab 2008 19 362 370 18938086 10.1016/j.tem.2008.08.007

[B91] Lin L Faraco J Li R Kadotani H Rogers W Lin X Qiu X de Jong PJ Nishino S Mignot E The sleep disorder canine narcolepsy is caused by a mutation in the hypocretin (orexin) receptor 2 gene Cell 1999 98 365 376 10458611 10.1016/s0092-8674(00)81965-0

[B92] Nishino S Ripley B Overeem S Lammers GJ Mignot E Hypocretin (orexin) deficiency in human narcolepsy Lancet 2000 355 39 40 10615891 10.1016/S0140-6736(99)05582-8

[B93] Hara J Beuckmann CT Nambu T Willie JT Chemelli RM Sinton CM Sugiyama F Yagami K Goto K Yanagisawa M Sakurai T Genetic ablation of orexin neurons in mice results in narcolepsy, hypophagia, and obesity Neuron 2001 30 345 354 11394998 10.1016/s0896-6273(01)00293-8

[B94] Roecker AJ Coleman PJ Orexin receptor antagonists: medicinal chemistry and therapeutic potential Curr Top Med Chem 2008 8 977 987 18673167 10.2174/156802608784936746

[B95] Coleman PJ Renger JJ Orexin receptor antagonists: a review of promising compounds patented since 2006 Expert Opin Ther Pat 2010 20 307 324 20180618 10.1517/13543770903567085

[B96] Coleman PJ Cox CD Roecker AJ Discovery of dual orexin receptor antagonists (DORAs) for the treatment of insomnia Curr Top Med Chem 2011 11 696 725 21261591 10.2174/1568026611109060696

[B97] Salomon RM Ripley B Kennedy JS Johnson B Schmidt D Zeitzer JM Nishino S Mignot E Diurnal variation of cerebrospinal fluid hypocretin-1 (Orexin-A) levels in control and depressed subjects Biol Psychiatry 2003 54 96 104 12873798 10.1016/s0006-3223(02)01740-7

[B98] Hirayama J Sahar S Grimaldi B Tamaru T Takamatsu K Nakahata Y Sassone-Corsi P CLOCK-mediated acetylation of BMAL1 controls circadian function Nature 2007 450 1086 1090 18075593 10.1038/nature06394

[B99] Edgar RS Green EW Zhao Y van Ooijen G Olmedo M Qin X Xu Y Pan M Valekunja UK Feeney KA Maywood ES Hastings MH Baliga NS Merrow M Millar AJ Johnson CH Kyriacou CP O’Neill JS Reddy AB Peroxiredoxins are conserved markers of circadian rhythms Nature 2012 485 459 464 22622569 10.1038/nature11088 PMC3398137

[B100] Marcheva B Ramsey KM Buhr ED Kobayashi Y Su H Ko CH Ivanova G Omura C Mo S Vitaterna MH Lopez JP Philipson LH Bradfield CA Crosby SD JeBailey L Wang X Takahashi JS Bass J Disruption of the clock components CLOCK and BMAL1 leads to hypoinsulinaemia and diabetes Nature 2010 466 627 631 20562852 10.1038/nature09253 PMC2920067

[B101] Turek FW Circadian clocks: tips from the tip of the iceberg Nature 2008 456 881 883 19092918 10.1038/456881a

[B102] Lamia KA Evans RM Metabolism: tick, tock, a [beta]-cell clock Nature 2010 466 571 572 20671699 10.1038/466571a PMC3610560

[B103] Rouyer F Circadian rhythms: no lazing on sunny afternoons Nature 2012 484 325 326 22517159 10.1038/484325a

[B104] Masri S Sassone-Corsi P Plasticity and specificity of the circadian epigenome Nat Neurosci 2010 13 1324 1329 20975756 10.1038/nn.2668 PMC4071955

[B105] Roth TL Sweatt JD Rhythms of memory Nat Neurosci 2008 11 993 994 18725902 10.1038/nn0908-993

[B106] Kessler RC Berglund P Demler O Jin R Merikangas KR Walters EE Lifetime prevalence and age-of-onset distributions of DSM-IV disorders in the national comorbidity survey replication Arch Gen Psychiatry 2005 62 593 602 15939837 10.1001/archpsyc.62.6.593

[B107] Avenevoli S Merikangas KR Implications of high-risk family studies for prevention of depression Am J Prev Med 2006 31 126 135 10.1016/j.amepre.2006.07.003 17175407

[B108] Wright MJ Martin NG Brisbane adolescent twin study: outline of study methods and research projects Aust J Psychol 2004 56 65 78

[B109] Hansell NK Wright MJ Medland SE Davenport TA Wray NR Martin NG Hickie IB Genetic co-morbidity between neuroticism, anxiety/depression and somatic distress in a population sample of adolescent and young adult twins Psychol Med 2012 42 1249 1260 22051348 10.1017/S0033291711002431

[B110] Hatton SN Lagopoulos J Hermens DF Naismith SL Bennett MR Hickie IB Correlating anterior insula gray matter volume changes in young people with clinical and neurocognitive outcomes: an MRI study BMC Psychiatry 2012 12 45 22607202 10.1186/1471-244X-12-45 PMC3468394

[B111] Lagopoulos J Hermens DF Naismith SL Scott EM Hickie IB Frontal lobe changes occur early in the course of affective disorders in young people BMC Psychiatry 2012 12 4 22264318 10.1186/1471-244X-12-4 PMC3280164

[B112] Gillespie NA Kirk KM Evans DM Heath AC Hickie IB Martin NG Do the genetic or environmental determinants of anxiety and depression change with age? A longitudinal study of Australian twins Twin Res 2004 7 39 53 15053853 10.1375/13690520460741435

[B113] Hickie I Scott E Wilhelm K Brodaty H Subcortical hyperintensities on magnetic resonance imaging in patients with severe depression–a longitudinal evaluation Biol Psychiatry 1997 42 367 374 9276077 10.1016/S0006-3223(96)00363-0

[B114] Hickie I Simons L Naismith S Simons J McCallum J Pearson K Vascular risk to late-life depression: evidence from a longitudinal community study Aust N Z J Psychiatry 2003 37 62 65 12534658 10.1046/j.1440-1614.2003.01105.x

[B115] Hickie I Scott E Naismith S Ward PB Turner K Parker G Mitchell P Wilhelm K Late-onset depression: genetic, vascular and clinical contributions Psychol Med 2001 31 1403 1412 11722155 10.1017/s0033291701004731

[B116] Naismith SL Norrie L Lewis SJ Rogers NL Scott EM Hickie IB Does sleep disturbance mediate neuropsychological functioning in older people with depression? J Affect Disord 2009 116 139 143 19128840 10.1016/j.jad.2008.11.017

[B117] Naismith SL Glozier N Burke D Carter PE Scott E Hickie IB Early intervention for cognitive decline: is there a role for multiple medical or behavioural interventions? Early Interv Psychiatry 2009 3 19 27 21352171 10.1111/j.1751-7893.2008.00102.x

[B118] Alexopoulos GS Schultz SK Lebowitz BD Late-life depression: a model for medical classification Biol Psychiatry 2005 58 283 289 16026764 10.1016/j.biopsych.2005.04.055 PMC7124284

[B119] Krishnan KR Hays JC Tupler LA George LK Blazer DG Clinical and phenomenological comparisons of late-onset and early-onset depression Am J Psychiatry 1995 152 785 788 7726320 10.1176/ajp.152.5.785

[B120] Naismith SL Diamond K Carter PE Norrie LM Redoblado-Hodge MA Lewis SJ Hickie IB Enhancing memory in late-life depression: the effects of a combined psychoeducation and cognitive training program Am J Geriatr Psychiatry 2010 19 240 248 20808114 10.1097/JGP.0b013e3181dba587

[B121] Christensen H Pallister E Smale S Hickie IB Calear AL Community-based prevention programs for anxiety and depression in youth: a systematic review J Prim Prev 2010 31 139 170 20437102 10.1007/s10935-010-0214-8

[B122] Paus T Keshavan M Giedd JN Why do many psychiatric disorders emerge during adolescence? Nat Rev Neurosci 2008 9 947 957 19002191 10.1038/nrn2513 PMC2762785

[B123] Robillard R Naismith SL Rogers NL Ip TK Hermens DF Scott EM Hickie IB Delayed sleep phase in young people with unipolar or bipolar affective disorders J Affect Disord 2013 145 260 263 22877966 10.1016/j.jad.2012.06.006

[B124] Merikangas KR Cui L Kattan G Carlson GA Youngstrom EA Angst J Mania with and without depression in a community sample of U.S. adolescents Arch Gen Psychiatry 2012 69 943 951 22566563 10.1001/archgenpsychiatry.2012.38 PMC11955849

[B125] Kirk KM Hickie IB Martin NG Fatigue as related to anxiety and depression in a community-based sample of twins aged over 50 Soc Psychiatry Psychiatr Epidemiol 1999 34 85 90 10189814 10.1007/s001270050116

[B126] Hickie I Kirk K Martin N Unique genetic and environmental determinants of prolonged fatigue: a twin study Psychol Med 1999 29 259 268 10218917 10.1017/s0033291798007934

[B127] Hickie I Bennett B Lloyd A Heath A Martin N Complex genetic and environmental relationships between psychological distress, fatigue and immune functioning: a twin study Psychol Med 1999 29 269 277 10218918 10.1017/s0033291798007922

[B128] van der Linden G Chalder T Hickie I Koschera A Sham P Wessely S Fatigue and psychiatric disorder: different or the same? Psychol Med 1999 29 863 868 10473313 10.1017/s0033291799008697

[B129] Lloyd AR Hickie I Peterson PK Chronic fatigue syndrome: current concepts of pathogenesis and treatment Curr Clin Top Infect Dis 1999 19 135 159 10472484

[B130] Hickie I Koschera A Hadzi-Pavlovic D Bennett B Lloyd A The temporal stability and co-morbidity of prolonged fatigue: a longitudinal study in primary care Psychol Med 1999 29 855 861 10473312 10.1017/s0033291799008582

[B131] Perlis ML Giles DE Buysse DJ Tu X Kupfer DJ Self-reported sleep disturbance as a prodromal symptom in recurrent depression J Affect Disord 1997 42 209 212 9105962 10.1016/s0165-0327(96)01411-5

[B132] Buysse DJ Reynolds CF 3rd Monk TH Berman SR Kupfer DJ The Pittsburgh Sleep Quality Index: a new instrument for psychiatric practice and research Psychiatry Res 1989 28 193 213 2748771 10.1016/0165-1781(89)90047-4

[B133] Agargun MY Besiroglu L Cilli AS Gulec M Aydin A Inci R Selvi Y Nightmares, suicide attempts, and melancholic features in patients with unipolar major depression J Affect Disord 2007 98 3 267 270 16938351 10.1016/j.jad.2006.08.005

[B134] Ohayon MM Caulet M Philip P Guilleminault C Priest RG How sleep and mental disorders are related to complaints of daytime sleepiness Arch Intern Med 1997 157 2645 2652 9531234

[B135] Dryman A Eaton WW Affective symptoms associated with the onset of major depression in the community: findings from the US National Institute of Mental Health Epidemiologic Catchment Area Program Acta Psychiatr Scand 1991 84 1 1 5 1927557 10.1111/j.1600-0447.1991.tb01410.x

[B136] Benca RM Obermeyer WH Thisted RA Gillin JC Sleep and psychiatric disorders. A meta-analysis Arch Gen Psychiatry 1992 49 8 651 668 discussion 669-670 1386215 10.1001/archpsyc.1992.01820080059010

[B137] Feinberg M Gillin JC Carroll BJ Greden JF Zis AP EEG studies of sleep in the diagnosis of depression Biol Psychiatry 1982 17 305 316 7082698

[B138] Thase ME Kupfer DJ Fasiczka AJ Buysse DJ Simons AD Frank E Identifying an abnormal electroencephalographic sleep profile to characterize major depressive disorder Biol Psychiatry 1997 41 964 973 9110102 10.1016/S0006-3223(96)00259-4

[B139] Kupfer DJ Foster FG Interval between onset of sleep and rapid-eye-movement sleep as an indicator of depression Lancet 1972 2 684 686 4115821 10.1016/s0140-6736(72)92090-9

[B140] Buysse DJ Frank E Lowe KK Cherry CR Kupfer DJ Electroencephalographic sleep correlates of episode and vulnerability to recurrence in depression Biol Psychiatry 1997 41 4 406 418 9034535 10.1016/S0006-3223(96)00041-8

[B141] Peppard PE Szklo-Coxe M Hla KM Young T Longitudinal association of sleep-related breathing disorder and depression Arch Intern Med 2006 166 16 1709 1715 16983048 10.1001/archinte.166.16.1709

[B142] Schroder CM O'Hara R Depression and Obstructive Sleep Apnea (OSA) Annals of general psychiatry 2005 4 13 15982424 10.1186/1744-859X-4-13 PMC1181621

[B143] Wehr TA Wirz-Justice A Goodwin FK Duncan W Gillin JC Phase advance of the circadian sleep-wake cycle as an antidepressant Science 1979 206 4419 710 713 227056 10.1126/science.227056

[B144] Nair NP Hariharasubramanian N Pilapil C Circadian rhythm of plasma melatonin in endogenous depression Prog Neuropsychopharmacol Biol Psychiatry 1984 8 4–6 715 718 6531443 10.1016/0278-5846(84)90044-7

[B145] Claustrat B Chazot G Brun J Jordan D Sassolas G A chronobiological study of melatonin and cortisol secretion in depressed subjects: plasma melatonin, a biochemical marker in major depression Biol Psychiatry 1984 19 8 1215 1228 6498244

[B146] Beck-Friis J Kjellman BF Aperia B Unden F von Rosen D Ljunggren JG Wetterberg L Serum melatonin in relation to clinical variables in patients with major depressive disorder and a hypothesis of a low melatonin syndrome Acta Psychiatr Scand 1985 71 4 319 330 4039876 10.1111/j.1600-0447.1985.tb02531.x

[B147] Souetre E Salvati E Belugou JL Pringuey D Candito M Krebs B Ardisson JL Darcourt G Circadian rhythms in depression and recovery: evidence for blunted amplitude as the main chronobiological abnormality Psychiatry Res 1989 28 3 263 278 2762432 10.1016/0165-1781(89)90207-2

[B148] Rao AV Devi SP Srinivasan V Urinary melatonin in depression Indian journal of psychiatry 1983 25 3 167 172 21847281 PMC3012312

[B149] Wetterberg L Aperia B Gorelick DA Gwirtzman HE McGuire MT Serafetinides EA Yuwiler A Age, alcoholism and depression are associated with low levels of urinary melatonin J Psychiatry Neurosci 1992 17 5 215 224 1489763 PMC1188459

[B150] Peeters F Nicolson NA Berkhof J Levels and variability of daily life cortisol secretion in major depression Psychiatry Res 2004 126 1 1 13 15081622 10.1016/j.psychres.2003.12.010

[B151] Linkowski P Mendlewicz J Leclercq R Brasseur M Hubain P Golstein J Copinschi G Van Cauter E The 24-hour profile of adrenocorticotropin and cortisol in major depressive illness J Clin Endocrinol Met 1985 61 429 438 10.1210/jcem-61-3-429 2991318

[B152] Avery DH Wildschiodtz G Rafaelsen OJ Nocturnal temperature in affective disorder J Affect Disord 1982 4 1 61 71 6461688 10.1016/0165-0327(82)90020-9

[B153] Taillard J Sanchez P Lemoine P Mouret J Heart rate circadian rhythm as a biological marker of desynchronization in major depression: a methodological and preliminary report Chronobiol Int 1990 7 4 305 316 2085871

[B154] Taillard J Lemoine P Boule P Drogue M Mouret J Sleep and heart rate circadian rhythm in depression: the necessity to separate Chronobiol Int 1993 10 1 63 72 8443845 10.3109/07420529309064483

[B155] Kario K Schwartz JE Davidson KW Pickering TG Gender differences in associations of diurnal blood pressure variation, awake physical activity, and sleep quality with negative affect: the work site blood pressure study Hypertension 2001 38 5 997 1002 11711488 10.1161/hy1101.095009

[B156] Alesci S Martinez PE Kelkar S Ilias I Ronsaville DS Listwak SJ Ayala AR Licinio J Gold HK Kling MA Chrousos GP Gold PW Major depression is associated with significant diurnal elevations in plasma interleukin-6 levels, a shift of its circadian rhythm, and loss of physiological complexity in its secretion: clinical implications J Clin Endocrinol Metab 2005 90 2522 2530 15705924 10.1210/jc.2004-1667

[B157] Bridges PK Jones MT The diurnal rhythm of plasma cortisol concentration in depression Br J Psychiatry 1966 112 493 1257 1261 5966155 10.1192/bjp.112.493.1257

[B158] Board F Wadeson R Persky H Depressive affect and endocrine functions; blood levels of adrenal cortex and thyroid hormones in patients suffering from depressive reactions AMA archives of neurology and psychiatry 1957 78 6 612 620 13478217

[B159] Mendlewicz J Linkowski P Branchey L Weinberg U Weitzman ED Branchey M Abnormal 24 hour pattern of melatonin secretion in depression Lancet 1979 2 8156–8157 1362 92694 10.1016/s0140-6736(79)92838-1

[B160] Weeke A Weeke J The 24-hour pattern of serum TSH in patients with endogenous depression Acta Psychiatr Scand 1980 62 1 69 74 7446193 10.1111/j.1600-0447.1980.tb00594.x

[B161] Brown R Kocsis JH Caroff S Amsterdam J Winokur A Stokes PE Frazer A Differences in nocturnal melatonin secretion between melancholic depressed patients and control subjects Am J Psychiatry 1985 142 7 811 816 4014502 10.1176/ajp.142.7.811

[B162] Crasson M Kjiri S Colin A Kjiri K L'Hermite-Baleriaux M Ansseau M Legros JJ Serum melatonin and urinary 6-sulfatoxymelatonin in major depression Psychoneuroendocrinology 2004 29 1 1 12 14575725 10.1016/s0306-4530(02)00123-3

[B163] Szymanska A Rabe-Jablonska J Karasek M Diurnal profile of melatonin concentrations in patients with major depression: relationship to the clinical manifestation and antidepressant treatment Neuro Endocrinol Lett 2001 22 3 192 198 11449197

[B164] Rabe-Jablonska J Szymanska A Diurnal profile of melatonin secretion in the acute phase of major depression and in remission Med Sci Monit 2001 7 5 946 952 11535940

[B165] Koenigsberg HW Teicher MH Mitropoulou V Navalta C New AS Trestman R Siever LJ 24-h Monitoring of plasma norepinephrine, MHPG, cortisol, growth hormone and prolactin in depression J Psychiatr Res 2004 38 5 503 511 15380401 10.1016/j.jpsychires.2004.03.006

[B166] Mendlewicz J Linkowski P Kerkhofs M Desmedt D Golstein J Copinschi G Van Cauter E Diurnal hypersecretion of growth hormone in depression J Clin Endocrinol Metabol 1985 60 3 505 512 10.1210/jcem-60-3-505 4038712

[B167] Branchey L Weinberg U Branchey M Linkowski P Mendlewicz J Simultaneous study of 24-hour patterns of melatonin and cortisol secretion in depressed patients Neuropsychobiology 1982 8 5 225 232 7133371 10.1159/000117903

[B168] Posener JA DeBattista C Williams GH Chmura Kraemer H Kalehzan BM Schatzberg AF 24-Hour monitoring of cortisol and corticotropin secretion in psychotic and nonpsychotic major depression Arch Gen Psychiatry 2000 57 8 755 760 10920463 10.1001/archpsyc.57.8.755

[B169] Gordijn MC Beersma DG Bouhuys AL Reinink E Van den Hoofdakker RH A longitudinal study of diurnal mood variation in depression; characteristics and significance J Affect Disord 1994 31 4 261 273 7989641 10.1016/0165-0327(94)90102-3

[B170] Germain A Nofzinger EA Meltzer CC Wood A Kupfer DJ Moore RY Buysse DJ Diurnal variation in regional brain glucose metabolism in depression Biol Psychiatry 2007 62 5 438 445 17217926 10.1016/j.biopsych.2006.09.043 PMC3195370

[B171] Joyce PR Porter RJ Mulder RT Luty SE McKenzie JM Miller AL Kennedy MA Reversed diurnal variation in depression: associations with a differential antidepressant response, tryptophan: large neutral amino acid ratio and serotonin transporter polymorphisms Psychol Med 2005 35 4 511 517 15856721 10.1017/s0033291704003861

[B172] Shin K Schaffer A Levitt AJ Boyle MH Seasonality in a community sample of bipolar, unipolar and control subjects J Affect Disord 2005 86 1 19 25 15820267 10.1016/j.jad.2004.11.010

[B173] Hasler BP Buysse DJ Kupfer DJ Germain A Phase relationships between core body temperature, melatonin, and sleep are associated with depression severity: further evidence for circadian misalignment in non-seasonal depression Psychiatry Res 2010 178 1 205 207 20471106 10.1016/j.psychres.2010.04.027 PMC2914120

[B174] Puig-Antich J Goetz R Hanlon C Davies M Thompson J Chambers WJ Tabrizi MA Weitzman ED Sleep architecture and REM sleep measures in prepubertal children with major depression: a controlled study Arch Gen Psychiatry 1982 39 932 939 7103682 10.1001/archpsyc.1982.04290080046007

[B175] Bertocci MA Dahl RE Williamson DE Iosif AM Birmaher B Axelson D Ryan ND Subjective sleep complaints in pediatric depression: a controlled study and comparison with EEG measures of sleep and waking J Am Acad Child Adolesc Psychiatry 2005 44 1158 1166 16239865 10.1097/01.chi.0000179057.54419.17

[B176] Armitage R Hoffmann R Emslie G Rintelmann J Robert J Sleep microarchitecture in childhood and adolescent depression: temporal coherence Clin EEG Neurosci 2006 37 1 1 9 16475478 10.1177/155005940603700103

[B177] Robert JJ Hoffmann RF Emslie GJ Hughes C Rintelmann J Moore J Armitage R Sex and age differences in sleep macroarchitecture in childhood and adolescent depression Sleep 2006 29 3 351 358 16553021 10.1093/sleep/29.3.351

[B178] Arana-Lechuga Y Nunez-Ortiz R Teran-Perez G Castillo-Montoya C Jimenez-Anguiano A Gonzalez-Robles RO Castro-Roman R Velazquez-Moctezuma J Sleep-EEG patterns of school children suffering from symptoms of depression compared to healthy controls World J Biol Psychiatry 2008 9 2 115 120 17853292 10.1080/15622970701216665

[B179] Emslie GJ Rush AJ Weinberg WA Rintelmann JW Roffwarg HP Sleep EEG features of adolescents with major depression Biol Psychiatry 1994 36 9 573 581 7833421 10.1016/0006-3223(94)90067-1

[B180] Goetz RR Puig-Antich J Ryan N Rabinovich H Ambrosini PJ Nelson B Krawiec V Electroencephalographic sleep of adolescents with major depression and normal controls Arch Gen Psychiatry 1987 44 1 61 68 3800585 10.1001/archpsyc.1987.01800130069009

[B181] Appelboom-Fondu J Kerkhofs M Mendlewicz J Depression in adolescents and young adults–polysomnographic and neuroendocrine aspects J Affect Disord 1988 14 1 35 40 2963050 10.1016/0165-0327(88)90069-9

[B182] Kutcher S Williamson P Marton P Szalai J REM latency in endogenously depressed adolescents Br J Psychiatry 1992 161 399 402 1393312 10.1192/bjp.161.3.399

[B183] Lahmeyer HW Poznanski EO Bellur SN EEG sleep in depressed adolescents Am J Psychiatry 1983 140 9 1150 1153 6614218 10.1176/ajp.140.9.1150

[B184] Rao U Poland RE Electroencephalographic sleep and hypothalamic-pituitary-adrenal changes from episode to recovery in depressed adolescents J Child Adolesc Psychopharmacol 2008 18 6 607 613 19108665 10.1089/cap.2008.034 PMC2672202

[B185] Armitage R Hoffmann R Emslie G Rintelman J Moore J Lewis K Rest-activity cycles in childhood and adolescent depression J Am Acad Child Adolesc Psychiatry 2004 43 6 761 769 15167093 10.1097/01.chi.0000122731.72597.4e

[B186] Dahl RE Ryan ND Puig-Antich J Nguyen NA Al-Shabbout M Meyer VA Perel J 24-hour cortisol measures in adolescents with major depression: a controlled study Biol Psychiatry 1991 30 1 25 36 1892959 10.1016/0006-3223(91)90067-v

[B187] Goodyer IM Herbert J Altham PM Pearson J Secher SM Shiers HM Adrenal secretion during major depression in 8- to 16-year-olds, I. Altered diurnal rhythms in salivary cortisol and dehydroepiandrosterone (DHEA) at presentation Psychol Med 1996 26 2 245 256 8685281 10.1017/s0033291700034644

[B188] Gillin JC Duncan WC Murphy DL Post RM Wehr TA Goodwin FK Wyatt RJ Bunney WE Jr Age-related changes in sleep in depressed and normal subjects Psychiatry Res 1981 4 1 73 78 6939001 10.1016/0165-1781(81)90010-x

[B189] Tuunainen A Kripke DF Elliott JA Assmus JD Rex KM Klauber MR Langer RD Depression and endogenous melatonin in postmenopausal women J Affect Disord 2002 69 1–3 149 158 12103461 10.1016/s0165-0327(01)00303-2

[B190] Hamada T Murata T Omori M Takahashi T Kosaka H Wada Y Yoshida H Abnormal nocturnal blood pressure fall in senile-onset depression with subcortical silent cerebral infarction Neuropsychobiology 2003 47 4 187 191 12824741 10.1159/000071213

[B191] Bunney WE Jr Murphy DL Goodwin FK Borge GF The switch process from depression to mania: relationship to drugs which alter brain amines Lancet 1970 1 1022 1027 4191630 10.1016/s0140-6736(70)91151-7

[B192] Sitaram N Gillin JC Bunney WE Jr The switch process in manic-depressive illness. Circadian variation in time of switch and sleep and manic ratings before and after switch Acta Psychiatr Scand 1978 58 267 278 707167 10.1111/j.1600-0447.1978.tb06938.x

[B193] Detre T Himmelhoch J Swartzburg M Anderson CM Byck R Kupfer DJ Hypersomnia and manic-depressive disease Am J Psychiatry 1972 128 1303 1305 4335278 10.1176/ajp.128.10.1303

[B194] Casper RC Redmond DE Jr Katz MM Schaffer CB Davis JM Koslow SH Somatic symptoms in primary affective disorder. Presence and relationship to the classification of depression Arch Gen Psychiatry 1985 42 1098 1104 3863548 10.1001/archpsyc.1985.01790340082012

[B195] Parker G Malhi G Hadzi-Pavlovic D Parker K Sleeping in? The impact of age and depressive sub-type on hypersomnia J Affect Disord 2006 90 1 73 76 16325918 10.1016/j.jad.2005.10.004

[B196] Goodwin FK Jamison KR Manic-Depressive Illness: Bipolar Disorders and Recurrent Depression 2007 New York: Oxford Univeristy Press

[B197] de Maertelaer V Hoffman G Lemaire M Mendlewicz J Sleep spindle activity changes in patients with affective disorders Sleep 1987 10 443 451 3685752 10.1093/sleep/10.5.443

[B198] Lauer CJ Wiegand M Krieg JC All-night electroencephalographic sleep and cranial computed tomography in depression. A study of unipolar and bipolar patients Eur Arch Psychiatry Clin Neurosci 1992 242 59 68 1486107 10.1007/BF02191547

[B199] Thase ME Himmelhoch JM Mallinger AG Jarrett DB Kupfer DJ Sleep EEG and DST findings in anergic bipolar depression Am J Psychiatry 1989 146 329 333 2919689 10.1176/ajp.146.3.329

[B200] Duncan WC Jr Pettigrew KD Gillin JC REM architecture changes in bipolar and unipolar depression Am J Psychiatry 1979 136 1424 1427 227281 10.1176/ajp.136.11.1424

[B201] Hudson JI Lipinski JF Frankenburg FR Grochocinski VJ Kupfer DJ Electroencephalographic sleep in mania Arch Gen Psychiatry 1988 45 267 273 3341881 10.1001/archpsyc.1988.01800270085010

[B202] Hudson JI Lipinski JF Keck PE Jr Aizley HG Lukas SE Rothschild AJ Waternaux CM Kupfer DJ Polysomnographic characteristics of young manic patients. Comparison with unipolar depressed patients and normal control subjects Arch Gen Psychiatry 1992 49 378 383 1586273 10.1001/archpsyc.1992.01820050042006

[B203] Kripke DF Mullaney DJ Atkinson M Wolf S Circadian rhythm disorders in manic-depressives Biol Psychiatry 1978 13 335 351 667233

[B204] Lewy AJ Nurnberger JI Jr Wehr TA Pack D Becker LE Powell RL Newsome DA Supersensitivity to light: possible trait marker for manic-depressive illness Am J Psychiatry 1985 142 725 727 4003592 10.1176/ajp.142.6.725

[B205] Lewy AJ Wehr TA Goodwin FK Newsome DA Rosenthal NE Manic-depressive patients may be supersensitive to light Lancet 1981 1 383 384 6110011 10.1016/s0140-6736(81)91697-4

[B206] Feldman-Naim S Turner EH Leibenluft E Diurnal variation in the direction of mood switches in patients with rapid-cycling bipolar disorder J Clin Psychiatry 1997 58 2 79 84 9062377 10.4088/jcp.v58n0205

[B207] Souetre E Salvati E Wehr TA Sack DA Krebs B Darcourt G Twenty-four-hour profiles of body temperature and plasma TSH in bipolar patients during depression and during remission and in normal control subjects Am J Psychiatry 1988 145 1133 1137 3414857 10.1176/ajp.145.9.1133

[B208] Thompson C Isaacs G Seasonal affective disorder–a British sample. Symptomatology in relation to mode of referral and diagnostic subtype J Affect Disord 1988 14 1 1 11 2963046 10.1016/0165-0327(88)90065-1

[B209] Giglio LM Magalhaes PV Andersen ML Walz JC Jakobson L Kapczinski F Circadian preference in bipolar disorder Sleep Breath 2010 14 2 153 155 19774406 10.1007/s11325-009-0301-3

[B210] Mansour HA Wood J Chowdari KV Dayal M Thase ME Kupfer DJ Monk TH Devlin B Nimgaonkar VL Circadian phase variation in bipolar I disorder Chronobiol Int 2005 22 3 571 584 16076655 10.1081/CBI-200062413

[B211] Robillard R Naismith SL Rogers NL Ip YKC Hermens DF Scott EM Hickie IB Delayed circadian rhythms in young persons with unipolar or bipolar affective disorders Proceedings of the 24th Annual Scientific meeting of the of Australasian Sleep Association and Australasian Sleep Technologists Association: 11-13 October 2012; Darwin. Sleep and Biological Rhythms 2012; 10 (Suppl. 1): 39

[B212] Saeed SA Bruce TJ Seasonal affective disorders Am Fam Physician 1998 57 1340 1346 9531916

[B213] Rosenthal NE Sack DA Gillin JC Lewy AJ Goodwin FK Davenport Y Mueller PS Newsome DA Wehr TA Seasonal affective disorder. A description of the syndrome and preliminary findings with light therapy Arch Gen Psychiatry 1984 41 72 80 6581756 10.1001/archpsyc.1984.01790120076010

[B214] Wehr TA Giesen HA Schulz PM Anderson JL Joseph-Vanderpool JR Kelly K Kasper S Rosenthal NE Contrasts between symptoms of summer depression and winter depression J Affect Disord 1991 23 173 183 1791262 10.1016/0165-0327(91)90098-d

[B215] Avery DH Dahl K Savage MV Brengelmann GL Larsen LH Kenny MA Eder DN Vitiello MV Prinz PN Circadian temperature and cortisol rhythms during a constant routine are phase-delayed in hypersomnic winter depression Biol Psychiatry 1997 41 1109 1123 9146822 10.1016/S0006-3223(96)00210-7

[B216] Dahl K Avery DH Lewy AJ Savage MV Brengelmann GL Larsen LH Vitiello MV Prinz PN Dim light melatonin onset and circadian temperature during a constant routine in hypersomnic winter depression Acta Psychiatr Scand 1993 88 60 66 8372697 10.1111/j.1600-0447.1993.tb03414.x

[B217] Lewy AJ Sack RL Singer CM White DM The phase shift hypothesis for bright light's therapeutic mechanism of action: theoretical considerations and experimental evidence Psychopharmacol Bull 1987 23 349 353 3324148

[B218] Thompson C Stinson D Fernandez M Fine J Isaacs G A comparison of normal, bipolar and seasonal affective disorder subjects using the Seasonal Pattern Assessment Questionnaire J Affect Disord 1988 14 3 257 264 2968387 10.1016/0165-0327(88)90043-2

[B219] Thompson C Stinson D Smith A Seasonal affective disorder and season-dependent abnormalities of melatonin suppression by light Lancet 1990 336 703 706 1975891 10.1016/0140-6736(90)92202-s

[B220] Akiskal HS Judd LL Gillin JC Lemmi H Subthreshold depressions: clinical and polysomnographic validation of dysthymic, residual and masked forms J Affect Disord 1997 45 53 63 9268775 10.1016/s0165-0327(97)00059-1

[B221] Zhou JN Riemersma RF Unmehopa UA Hoogendijk WJ van Heerikhuize JJ Hofman MA Swaab DF Alterations in arginine vasopressin neurons in the suprachiasmatic nucleus in depression Arch Gen Psychiatry 2001 58 655 662 11448372 10.1001/archpsyc.58.7.655

[B222] Parker G Hadzi-Pavlovic D Brodaty H Boyce P Mitchell P Wilhelm K Hickie I Eyers K Psychomotor disturbance in depression: defining the constructs J Affect Disord 1993 27 255 265 8509526 10.1016/0165-0327(93)90049-p

[B223] Parker G Hadzi-Pavlovic D Boyce P Wilhelm K Brodaty H Mitchell P Hickie I Eyers K Classifying depression by mental state signs Br J Psychiatry 1990 157 55 65 2397363 10.1192/bjp.157.1.55

[B224] Hickie I Mason C Parker G Comparative validity of two measures of psychomotor function in patients with severe depression J Affect Disord 1996 37 143 149 8731077 10.1016/0165-0327(95)00087-9

[B225] Schrijvers D Hulstijn W Sabbe BG Psychomotor symptoms in depression: a diagnostic, pathophysiological and therapeutic tool J Affect Disord 2008 109 1 20 18082896 10.1016/j.jad.2007.10.019

[B226] Sobin C Sackeim HA Psychomotor symptoms of depression Am J Psychiatry 1997 154 4 17 8988952 10.1176/ajp.154.1.4

[B227] Wulff K Porcheret K Cussans E Foster RG Sleep and circadian rhythm disturbances: multiple genes and multiple phenotypes Curr Op Genet Dev 2009 19 237 246 19423332 10.1016/j.gde.2009.03.007

[B228] Artioli P Lorenzi C Pirovano A Serretti A Benedetti F Catalano M Smeraldi E How do genes exert their role? Period 3 gene variants and possible influences on mood disorder phenotypes Eur Neuropsychopharmacol 2007 17 587 594 17512705 10.1016/j.euroneuro.2007.03.004

[B229] Kripke DF Nievergelt CM Joo E Shekhtman T Kelsoe JR Circadian polymorphisms associated with affective disorders J Circ Rhythms 2009 7 2 10.1186/1740-3391-7-2 PMC2661876 19166596

[B230] Severino G Manchia M Contu P Squassina A Lampus S Ardau R Chillotti C Del Zompo M Association study in a Sardinian sample between bipolar disorder and the nuclear receptor REV-ERBalpha gene, a critical component of the circadian clock system Bipolar Disord 2009 11 215 220 19267705 10.1111/j.1399-5618.2009.00667.x

[B231] McGrath CL Glatt SJ Sklar P Le-Niculescu H Kuczenski R Doyle AE Biederman J Mick E Faraone SV Niculescu AB Tsuang MT Evidence for genetic association of RORB with bipolar disorder BMC Psychiatry 2009 9 70 19909500 10.1186/1471-244X-9-70 PMC2780413

[B232] Nievergelt CM Kripke DF Barrett TB Burg E Remick RA Sadovnick AD McElroy SL Keck PE Jr Schork NJ Kelsoe JR Suggestive evidence for association of the circadian genes PERIOD3 and ARNTL with bipolar disorder Am J Med Genet B Neuropsychiatr Genet 2006 141B 234 241 16528748 10.1002/ajmg.b.30252 PMC2651679

[B233] Mansour HA Wood J Logue T Chowdari KV Dayal M Kupfer DJ Monk TH Devlin B Nimgaonkar VL Association study of eight circadian genes with bipolar I disorder, schizoaffective disorder and schizophrenia Genes Brain Behav 2006 5 150 157 16507006 10.1111/j.1601-183X.2005.00147.x

[B234] Soria V Martinez-Amoros E Escaramis G Valero J Perez-Egea R Garcia C Gutierrez-Zotes A Puigdemont D Bayes M Crespo JM Martorell L Vilella E Labad A Vallejo J Perez V Menchon JM Estivill X Gratacos M Urretavizcaya M Differential association of circadian genes with mood disorders: CRY1 and NPAS2 are associated with unipolar major depression and CLOCK and VIP with bipolar disorder Neuropsychopharmacology 2010 35 1279 1289 20072116 10.1038/npp.2009.230 PMC3055337

[B235] Benedetti F Barbini B Bernasconi A Fulgosi MC Dallaspezia S Gavinelli C Locatelli C Lorenzi C Pirovano A Radaelli D Acute antidepressant response to sleep deprivation combined with light therapy is influenced by the catechol-O-methyltransferase Val(108/158)Met polymorphism J Affect Disord 2010 121 1–2 68 72 19520435 10.1016/j.jad.2009.05.017

[B236] Naismith SL Lewis SJ Rogers NL Sleep-wake changes and cognition in neurodegenerative disease Prog Brain Res 2011 190 21 52 21531243 10.1016/B978-0-444-53817-8.00002-5

[B237] Valkanova V Ebmeier KP Vascular risk factors and depression in later life: a systematic review and meta-analysis Biol Psychiatry 2013 73 5 406 413 23237315 10.1016/j.biopsych.2012.10.028

[B238] Xekardaki A Santos M Hof P Kövari E Bouras C Giannakopoulos P Neuropathological substrates and structural changes in late-life depression: the impact of vascular burden Acta Neuropathol 2012 124 4 453 464 22836715 10.1007/s00401-012-1021-5

[B239] Norris ER Karen B Correll JR Zemanek KJ Lerman J Primelo RA Kaufmann MW A double-blind, randomized, placebo-controlled trial of adjunctive ramelteon for the treatment of insomnia and mood stability in patients with euthymic bipolar disorder J Affect Disord 2013 144 141 147 22963894 10.1016/j.jad.2012.06.023

[B240] Goetze U Tolle R Circadian rhythm of free urinary cortisol, temperature and heart rate in endogenous depressives and under antidepressant therapy Neuropsychobiology 1987 18 175 184 3454423 10.1159/000118414

[B241] Buhr ED Yoo SH Takahashi JS Temperature as a universal resetting cue for mammalian circadian oscillators Science 2010 330 379 385 20947768 10.1126/science.1195262 PMC3625727

[B242] Youngstedt SD Kripke DF Elliott JA Klauber MR Circadian abnormalities in older adults J Pineal Res 2001 31 264 272 11589762 10.1034/j.1600-079x.2001.310311.x

[B243] Cole RJ Smith JS Alcala YC Elliott JA Kripke DF Bright-light mask treatment of delayed sleep phase syndrome J Biol Rhythms 2002 17 89 101 11837952 10.1177/074873002129002366

[B244] Nagtegaal JE Kerkhof GA Smits MG Swart AC van der Meer YG Traumatic brain injury-associated delayed sleep phase syndrome Funct Neurol 1997 12 345 348 9503198

[B245] Nagtegaal JE Kerkhof GA Smits MG Swart AC van der Meer YG Delayed sleep phase syndrome: a placebo-controlled cross-over study on the effects of melatonin administered five hours before the individual dim light melatonin onset J Sleep Res 1998 7 135 143 9682186 10.1046/j.1365-2869.1998.00102.x

[B246] Middleton B Arendt J Stone BM Complex effects of melatonin on human circadian rhythms in constant dim light J Biol Rhythms 1997 12 467 477 9376645 10.1177/074873049701200508

[B247] Middleton B Arendt J Stone BM Human circadian rhythms in constant dim light (8 lux) with knowledge of clock time J Sleep Res 1996 5 69 76 8795806 10.1046/j.1365-2869.1996.d01-67.x

[B248] Carskadon MA Acebo C Richardson GS Tate BA Seifer R An approach to studying circadian rhythms of adolescent humans J Biol Rhythms 1997 12 278 289 9181439 10.1177/074873049701200309

[B249] Carskadon MA Wolfson AR Acebo C Tzischinsky O Seifer R Adolescent sleep patterns, circadian timing, and sleepiness at a transition to early school days Sleep 1998 21 871 881 9871949 10.1093/sleep/21.8.871

[B250] Serre F Fatseas M Debrabant R Alexandre JM Auriacombe M Swendsen J Ecological momentary assessment in alcohol, tobacco, cannabis and opiate dependence: a comparison of feasibility and validity Drug Alcohol Depend 2012 126 118 123 22647899 10.1016/j.drugalcdep.2012.04.025

[B251] Granholm E Loh C Swendsen J Feasibility and validity of computerized ecological momentary assessment in schizophrenia Schizophr Bull 2008 34 507 514 17932087 10.1093/schbul/sbm113 PMC2632427

[B252] Duffy A Alda M Crawford L Milin R Grof P The early manifestations of bipolar disorder: a longitudinal prospective study of the offspring of bipolar parents Bipolar Disord 2007 9 828 838 18076532 10.1111/j.1399-5618.2007.00421.x

[B253] Gregory AM Rijsdijk FV Lau JY Dahl RE Eley TC The direction of longitudinal associations between sleep problems and depression symptoms: a study of twins aged 8 and 10 years Sleep 2009 32 189 199 19238806 10.1093/sleep/32.2.189 PMC2635583

[B254] Buysse DJ Angst J Gamma A Ajdacic V Eich D Rossler W Prevalence, course, and comorbidity of insomnia and depression in young adults Sleep 2008 31 473 480 18457234 10.1093/sleep/31.4.473 PMC2279748

[B255] Roane BM Taylor DJ Adolescent insomnia as a risk factor for early adult depression and substance abuse Sleep 2008 31 1351 1356 18853932 PMC2572740

[B256] Rao U Dahl RE Ryan ND Birmaher B Williamson DE Rao R Kaufman J Heterogeneity in EEG sleep findings in adolescent depression: unipolar versus bipolar clinical course J Affect Disord 2002 70 273 280 12128239 10.1016/s0165-0327(01)00396-2

[B257] Naismith SL Rogers NL Hickie IB Mackenzie J Norrie LM Lewis SJ Sleep well, think well: sleep-wake disturbance in mild cognitive impairment J Geriatr Psychiatry Neurol 2010 23 123 130 20354239 10.1177/0891988710363710

[B258] Naismith SL Rogers NL Mackenzie J Hickie IB Lewis SJ The relationship between actigraphically defined sleep disturbance and REM sleep behaviour disorder in parkinson's disease Clin Neurol Neurosurg 2010 112 420 423 20303647 10.1016/j.clineuro.2010.02.011

[B259] Naismith SL Rogers NL Lewis SJ Diamond K Terpening Z Norrie L Hickie IB Sleep disturbance in mild cognitive impairment: differential effects of current and remitted depression Acta Neuropsychiatrica 2011 23 167 172 10.1111/j.1601-5215.2011.00555.x 25379794

[B260] Naismith SL Terpening Z Shine JM Lewis SJ Neuropsychological functioning in Pparkinson’s disease: differential relationships with self-reported sleep-wake disturbances Mov Disord 2011 26 1537 1541 21469205 10.1002/mds.23640

[B261] Ellis P Royal Australian and New Zealand College of Psychiatrists Clinical Practice Guidelines Team for Depression Australian and New Zealand clinical practice guideline for the treatment of depression Aust NZ J Psychiatry 2004 38 389 407 10.1080/j.1440-1614.2004.01377.x 15209830

[B262] Whitty P Gilbody S NICE, but will they help people with depression? The new national institute for clinical excellence depression guidelines Br J Psychiatry 2005 186 177 178 15738494 10.1192/bjp.186.3.177

[B263] Bunney BG Bunney WE Rapid-acting antidepressant strategies: mechanisms of action Int J Neuropsychopharmacol 2012 15 695 713 21733282 10.1017/S1461145711000927

[B264] Bunney JN Potkin SG Circadian abnormalities, molecular clock genes and chronobiological treatments in depression Br Med Bull 2008 86 1 23 32 18487629 10.1093/bmb/ldn019

[B265] Ehlers CL Kupfer DJ Frank E Monk TH Biological rhythms and depression: the role of zeitgebers and zeitstoreres Depression 1993 1 285 293

[B266] Bootzin RR Stimulus control treatment for Insomnia80th Annual Convention of the American Psychological Association 1972 Honolulu, HI 395 396

[B267] Spielman AJ Saskin P Thorpy MJ Treatment of chronic insomnia by restriction of time in bed Sleep 1987 10 1 45 56 3563247

[B268] Shoham-Salomon V Rosenthal R Paradoxical interventions: a meta-analysis J Consult Clin Psychol 1987 55 1 22 28 3571654 10.1037//0022-006x.55.1.22

[B269] Harvey AG Payne S The management of unwanted pre-sleep thoughts in insomnia: distraction with imagery versus general distraction Behav Res Ther 2002 40 3 267 277 11863237 10.1016/s0005-7967(01)00012-2

[B270] Buysse DJPMM The evaluation and treatment of insomnia Journal of Practical Psychiatry & Behavioral Health 1996 2 2 80 93

[B271] Wirz-Justice A Benedetti F Terman M Chronotherapeutics for Affective Disorders: A Clinician’s Manual for Light and Wake Therapy 2009 New York, NY: S Karger 10.1017/s003329170500437x 16045060

[B272] Czeisler CA Richardson GS Coleman RM Zimmerman JC Moore-Ede MC Dement WC Weitzman ED Chronotherapy: resetting the circadian clocks of patients with delayed sleep phase insomnia Sleep 1981 4 1 1 21 7232967 10.1093/sleep/4.1.1

[B273] Terman M Terman JS Light therapy for seasonal and nonseasonal depression: efficacy, protocol, safety, and side effects CNS Spectr 2005 10 647 663 quiz 672 16041296 10.1017/s1092852900019611

[B274] Barbini B Benedetti F Colombo C Dotoli D Bernasconi A Cigala-Fulgosi M Florita M Smeraldi E Dark therapy for mania: a pilot study Bipolar Disord 2005 7 98 101 15654938 10.1111/j.1399-5618.2004.00166.x

[B275] Wehr TA Sack DA Rosenthal NE Antidepressant effects of sleep deprivation and phototherapy Acta Psychiatr Belg 1985 85 5 593 602 4091021

[B276] Chesson AL Jr Littner M Davila D Anderson WM Grigg-Damberger M Hartse K Johnson S Wise M Practice parameters for the use of light therapy in the treatment of sleep disorders. Standards of practice committee, american academy of sleep medicine Sleep 1999 22 5 641 660 10450599 10.1093/sleep/22.5.641

[B277] Czeisler CA Kronauer RE Mooney JJ Anderson JL Allan JS Biologic rhythm disorders, depression, and phototherapy. A new hypothesis Psychiatr Clin North Am 1987 10 4 687 709 3332326

[B278] Wirz-Justice A Beginning to see the light Arch Gen Psychiatry 1998 55 10 861 862 9783554 10.1001/archpsyc.55.10.861

[B279] Jindal RD Thase ME Treatment of insomnia associated with clinical depression Sleep Med Rev 2004 8 19 30 15062208 10.1016/S1087-0792(03)00025-X

[B280] Winokur A Gary KA Rodner S Rae-Red C Fernando AT Szuba MP Depression, sleep physiology, and antidepressant drugs Depress Anxiety 2001 14 19 28 11568979 10.1002/da.1043

[B281] Wilson S Argyropoulos S Antidepressants and sleep: a qualitative review of the literature Drugs 2005 65 927 947 15892588 10.2165/00003495-200565070-00003

[B282] Gursky JT Krahn LE The effects of antidepressants on sleep: a review Harv Rev Psychiatry 2000 8 298 306 11133824

[B283] Wirz-Justice A Graw P Krauchi K Gisin B Arendt J Aldhous M Poldinger W Morning or nighttime melatonin is ineffective in seasonal affective disorder J Psychiatr Res 1990 24 129 137 2213636 10.1016/0022-3956(90)90053-s

[B284] Leibenluft E Feldman-Naim S Turner EH Wehr TA Rosenthal NE Effects of exogenous melatonin administration and withdrawal in five patients with rapid-cycling bipolar disorder J Clin Psychiatry 1997 58 383 388 9378688 10.4088/jcp.v58n0902

[B285] Dolberg OT Hirschmann S Grunhaus L Melatonin for the treatment of sleep disturbances in major depressive disorder Am J Psychiatry 1998 155 1119 1121 9699707 10.1176/ajp.155.8.1119

[B286] Morin CM Insomnia: Psychological Assessment and Management 1993 New York, NY: Guilford Press

[B287] Edinger JD Carney CE Overcoming Insomnia; A Cognitive-Behavioural Therapy Approach (Therapist Guide) 2008 New York, NY: Oxford University Press

[B288] Perlis ML Jungquist C Smith MT Posner D Cognitive Behavioral Treatment of Insomnia: A Session-by-Session Guide 2005 New York, NY: Springer

[B289] Wirz-Justice A Van den Hoofdakker RH Sleep deprivation in depression: what do we know, where do we go? Biol Psychiatry 1999 46 4 445 453 10459393 10.1016/s0006-3223(99)00125-0

[B290] Pflug B The effect of sleep deprivation on depressed patients Acta Psychiatr Scand 1976 53 2 148 158 1251760 10.1111/j.1600-0447.1976.tb00068.x

[B291] Wehr TA Rosenthal NE Sack DA Gillin JC Antidepressant effects of sleep deprivation in bright and dim light Acta Psychiatr Scand 1985 72 2 161 165 4050508 10.1111/j.1600-0447.1985.tb02589.x

[B292] Harris J Lack L Kemp K Wright H Bootzin R A randomized controlled trial of intensive sleep retraining (ISR): a brief conditioning treatment for chronic insomnia Sleep 2012 35 49 60 22215918 10.5665/sleep.1584 PMC3242687

[B293] Kahn M Baker BL Weiss JM Treatment of insomnia by relaxation training J Abnorm Psychol 1968 73 6 556 558 5717357 10.1037/h0026599

[B294] Bernstein DA Borkovec TD Progressive relaxation training: a manual for the helping professions. In., edn 1973 Michigan: Research Press

[B295] Smith J Advances in ABC relaxation: Application and inventories 2001 New York, NY: Springer

[B296] Schultz JH Luthe W Autogenic Therapy 1969 New York, NY: Grune and Stratton 1

[B297] Kupfer DJ Ehlers CL Frank E Grochocinski VJ McEachran AB Buhari A Persistent effects of antidepressants: EEG sleep studies in depressed patients during maintenance treatment Biol Psychiatry 1994 35 781 793 8043708 10.1016/0006-3223(94)91140-1

[B298] Ehlers CL Havstad JW Kupfer DJ Estimation of the time course of slow-wave sleep over the night in depressed patients: effects of clomipramine and clinical response Biol Psychiatry 1996 39 171 181 8837978 10.1016/0006-3223(95)00139-5

[B299] Campbell SS Gillin JC Kripke DF Janowsky DS Risch SC Lithium delays circadian phase of temperature and REM sleep in a bipolar depressive: a case report Psychiatry Res 1989 27 23 29 2493655 10.1016/0165-1781(89)90005-x

[B300] Benedetti F Barbini B Campori E Fulgosi MC Pontiggia A Colombo C Sleep phase advance and lithium to sustain the antidepressant effect of total sleep deprivation in bipolar depression: new findings supporting the internal coincidence model? J Psychiatr Res 2001 35 6 323 329 11684139 10.1016/s0022-3956(01)00034-6

[B301] Abe M Herzog ED Block GD Lithium lengthens the circadian period of individual suprachiasmatic nucleus neurons Neuroreport 2000 11 14 3261 3264 11043560 10.1097/00001756-200009280-00042

[B302] Kaladchibachi SA Doble B Anthopoulos N Woodgett JR Manoukian AS Glycogen synthase kinase 3, circadian rhythms, and bipolar disorder: a molecular link in the therapeutic action of lithium J Circ Rhythms 2007 5 3 10.1186/1740-3391-5-3 PMC1803776 17295926

[B303] Wilson SJ Nutt DJ Alford C Argyropoulos SV Baldwin DS Bateson AN Britton TC Crowe C Dijk DJ Espie CA Gringras P Hajak G Idzikowski C Krystal AD Nash JR Selsick H Sharpley AL Wade AG British Association for Psychopharmacology consensus statement on evidence-based treatment of insomnia, parasomnias and circadian rhythm disorders J Psychopharmacol 2010 24 1577 1601 20813762 10.1177/0269881110379307

[B304] Zhdanova IV Wurtman RJ Regan MM Taylor JA Shi JP Leclair OU Melatonin treatment for age-related insomnia J Clinical Endocrinol Metab 2001 86 4727 4730 11600532 10.1210/jcem.86.10.7901

[B305] Leger D Laudon M Zisapel N Nocturnal 6-sulfatoxymelatonin excretion in insomnia and its relation to the response to melatonin replacement therapy Am J Med 2004 116 91 95 14715322 10.1016/j.amjmed.2003.07.017

[B306] Rizvi SJ Kennedy SH The **keys to improving depression outcomes** Eur Neuropsychopharmacol 2011 21 Supplement 4 S694 S702 21924209 10.1016/j.euroneuro.2011.07.002

[B307] Pjrek E Winkler D Konstantinidis A Willeit M Praschak-Rieder N Kasper S Agomelatine in the treatment of seasonal affective disorder Psychopharmacology (Berl) 2007 190 575 579 17171557 10.1007/s00213-006-0645-3

[B308] Gorwood P Restoring circadian rhythms: a new way to successfully manage depression J Psychopharmacol 2010 24 15 19 20663804 10.1177/1359786810372981

[B309] Leproult R Van Onderbergen A L’Hermite-Balériaux M Van Cauter E Copinschi G Phase-shifts of 24-h rhythms of hormonal release and body temperature following early evening administration of the melatonin agonist agomelatine in healthy older men Clin Endocrinol 2005 63 298 304 10.1111/j.1365-2265.2005.02341.x 16117817

[B310] Quera Salva MA Vanier B Laredo J Hartley S Chapotot F Moulin C Lofaso F Guilleminault C Major depressive disorder, sleep EEG and agomelatine: an open-label study Int J Neuropsychopharmacol 2007 10 691 696 17477886 10.1017/S1461145707007754

[B311] Lemoine P Guilleminault C Alvarez E Improvement in subjective sleep in major depressive disorder with a novel antidepressant, agomelatine: randomized, double-blind comparison with venlafaxine J Clin Psychiatry 2007 68 1723 1732 18052566 10.4088/jcp.v68n1112

[B312] Miyamoto M Pharmacology of ramelteon, a selective MT1/MT2 receptor agonist: a novel therapeutic drug for sleep disorders CNS Neurosci Ther 2009 15 32 51 19228178 10.1111/j.1755-5949.2008.00066.x PMC2871175

[B313] Rajaratnam SM Polymeropoulos MH Fisher DM Roth T Scott C Birznieks G Klerman EB Melatonin agonist tasimelteon (VEC-162) for transient insomnia after sleep-time shift: two randomised controlled multicentre trials Lancet 2009 373 482 491 19054552 10.1016/S0140-6736(08)61812-7

[B314] Nickelsen T Samel A Vejvoda M Wenzel J Smith B Gerzer R Chronobiotic effects of the melatonin agonist LY 156735 following a simulated 9 h time shift: results of a placebo-controlled trial Chronobiol Int 2002 19 915 936 12405554 10.1081/cbi-120014108

[B315] Singh SP Singh V Kar N Efficacy of agomelatine in major depressive disorder: meta-analysis and appraisal Int J Neuropsychopharmacol 2012 13 417 428 21859514 10.1017/S1461145711001301

[B316] Barbui C Cipriani A Agomelatine and the brave old world of narrative-based medicine Evid Based Ment Health 2012 15 2 3 22252949 10.1136/ebmh.2011.100485

[B317] Montgomery SA Kasper S Severe depression and antidepressants: focus on a pooled analysis of placebo-controlled studies on agomelatine Int Clin Psychopharmacol 2007 22 283 291 17690597 10.1097/YIC.0b013e3280c56b13

[B318] Quera-Salva MA Lemoine P Guilleminault C Impact of the novel antidepressant agomelatine on disturbed sleep-wake cycles in depressed patients Hum Psychopharmacol 2010 25 222 229 20373473 10.1002/hup.1112

[B319] Sack RL Lewy AJ White DM Singer CM Fireman MJ Vandiver R Morning vs evening light treatment for winter depression. Evidence that the therapeutic effects of light are mediated by circadian phase shifts Arch Gen Psychiatry 1990 47 4 343 351 2322085 10.1001/archpsyc.1990.01810160043008

[B320] Wu JC Kelsoe JR Schachat C Bunney BG DeModena A Golshan S Gillin JC Potkin SG Bunney WE Rapid and sustained antidepressant response with sleep deprivation and chronotherapy in bipolar disorder Biol Psychiatry 2009 66 3 298 301 19358978 10.1016/j.biopsych.2009.02.018

[B321] Voderholzer U Valerius G Schaerer L Riemann D Giedke H Schwarzler F Berger M Wiegand M Is the antidepressive effect of sleep deprivation stabilized by a three day phase advance of the sleep period? A pilot study Eur Arch Psychiatry Clin Neurosci 2003 253 68 72 12799743 10.1007/s00406-003-0408-7

[B322] Souetre E Salvati E Pringuey D Plasse Y Savelli M Darcourt G Antidepressant effects of the sleep/wake cycle phase advance. Preliminary report J Affect Disord 2006 60 896 899 10.1016/0165-0327(87)90059-0 2952693

[B323] Sprouse J Braselton J Reynolds L Fluoxetine modulates the circadian biological clock via phase advances of suprachiasmatic nucleus neuronal firing Biol Psychiatry 2006 60 896 899 16631132 10.1016/j.biopsych.2006.03.003

[B324] Krauchi K Cajochen C Mori D Graw P Wirz-Justice A Early evening melatonin and S-20098 advance circadian phase and nocturnal regulation of core body temperature Am J Physiology 1997 272 R1178 R1188 10.1152/ajpregu.1997.272.4.R1178 9140018

[B325] McEachron DL Kripke DF Sharp FR Lewy AJ McClellan DE Lithium effects on selected circadian rhythms in rats Brain Res Bull 1985 15 347 350 4052829 10.1016/0361-9230(85)90162-5

[B326] Nagayama H Chronic administration of imipramine and lithium changes the phase-angle relationship between the activity and core body temperature circadian rhythms in rats Chronobiol Int 1996 13 251 259 8889249 10.3109/07420529609020905

[B327] Angst J The bipolar spectrum Br J Psychiatry 2007 190 189 191 17329735 10.1192/bjp.bp.106.030957

